# The *Helicobacter pylori* infection alters the intercellular junctions on the pancreas of gerbils (*Meriones unguiculatus*)

**DOI:** 10.1007/s11274-024-04081-0

**Published:** 2024-07-20

**Authors:** Edgar G. Hurtado-Monzón, Pedro Valencia-Mayoral, Angélica Silva-Olivares, Cecilia Bañuelos, Norma Velázquez-Guadarrama, Abigail Betanzos

**Affiliations:** 1 Departamento de Infectómica y Patogénesis Molecular, Centro de Investigación y de Estudios Avanzados del Instituto Politécnico Nacional (CINVESTAV-IPN), Ciudad de Mexico, México; 2 Departamento de Patología Clínica y Experimental del Hospital Infantil de México Federico Gómez, Ciudad de Mexico, México; 3 Programa de Doctorado Transdisciplinario en Desarrollo Científico y Tecnológico Para La Sociedad, CINVESTAV-IPN, Ciudad de Mexico, México; 4 Laboratorio de Investigación en Enfermedades Infecciosas, Área de Genética Bacteriana del Hospital Infantil de México Federico Gómez, Ciudad de Mexico, México

**Keywords:** Adherens junctions, Animal model, Bacterial infection, Desmosomes, Epithelial function, Inflammation, Tight junctions

## Abstract

*Helicobacter pylori* is a common resident in the stomach of at least half of the world’s population and recent evidence suggest its emergence in other organs such as the pancreas. In this organ, the presence of *H. pylori* DNA has been reported in cats, although the functional implications remain unknown. In this work, we determined distinct features related to the *H. pylori* manifestation in pancreas in a rodent model, in order to analyse its functional and structural effect. Gerbils inoculated with *H. pylori* exhibited the presence of this bacterium, as revealed by the expression of some virulence factors, as CagA and OMPs in stomach and pancreas, and confirmed by urease activity, bacterial culture, PCR and immunofluorescence assays. Non-apparent morphological changes were observed in pancreatic tissue of infected animals; however, delocalization of intercellular junction proteins (claudin-1, claudin-4, occludin, ZO-1, E-cadherin, β-catenin, desmoglein-2 and desmoplakin I/II) and rearrangement of the actin-cytoskeleton were exhibited. This structural damage was consistent with alterations in the distribution of insulin and glucagon, and a systemic inflammation, event demonstrated by elevated IL-8 levels. Overall, these findings indicate that *H. pylori* can reach the pancreas, possibly affecting its function and contributing to the development of pancreatic diseases.

**Graphical Abstract:**

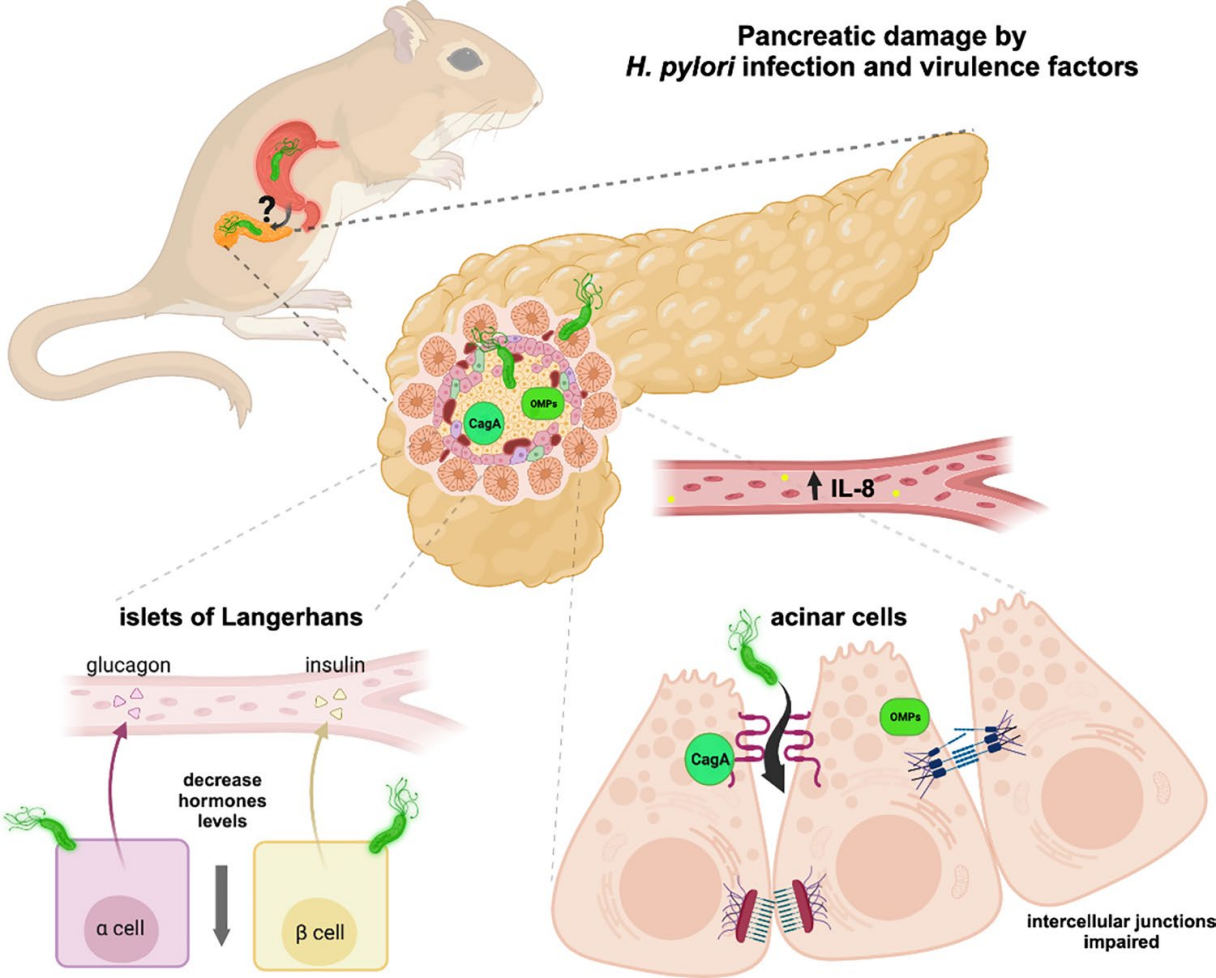

**Supplementary Information:**

The online version contains supplementary material available at 10.1007/s11274-024-04081-0.

## Introduction

*Helicobacter pylori* is a Gram-negative bacterium that infects at least half of the world’s population (Malfertheiner et al. 2023). Actually, it is the most common infectious pathogen of the gastroduodenal tract. Different factors, such as geographical region, host age, and human socioeconomic and hygienic aspects influence the prevalence or incidence of *H. pylori* infection (Hooi et al. 2017). The infection is acquired by oral ingestion of contaminated food, and mainly transmitted within families in early childhood (Suerbaum and Michetti 2002). After being ingested, *H. pylori* evades the bactericidal activity of the gastric luminal content and enters the mucous layer. There, the bacterial urease hydrolyses urea into carbon dioxide and ammonia, allowing *H. pylori* to survive in the acidic milieu (Mobley 2001). In addition, motility is essential for colonization, thereby, the presence of flagella contributes to bacterial adaptation to the gastric niche (Lertsethtakarn et al. 2011). Most *H. pylori* strains possess the cag pathogenicity island (*cag*-PAI), a 37-kb genomic region encompassing 29 genes. Many of them encode components of the type IV secretion system that translocates the CagA protein into the host cell cytoplasm. When CagA enters the epithelial cell, it is phosphorylated and attaches to the SHP-2 tyrosine phosphatase, which causes the host cell to produce growth factor–like cellular response and cytokines (Suerbaum and Michetti 2002). Other molecules also contribute to pathogenicity, including adhesins (e.g., SabA and BabA), outer membrane proteins (OMPs), serine proteases (HtrA) and the vacuolating cytotoxin A (VacA) (Matsuo et al. 2017; Chauhan et al. 2019; Doohan et al. 2021).

*H. pylori* produces continuous gastric inflammation in all infected people (Suerbaum and Michetti 2002). Chronic gastritis strongly correlates with the risk of clinical sequelae, such as mucosal atrophy, gastric or duodenal ulcers, or carcinoma and lymphoma in stomach (Suerbaum and Michetti 2002). In addition to colonizing the stomach, evidences suggest the presence of *H. pylori* in other organs such as the intestine, liver, glad bladder, coronary arteries, and pancreas (Nilsson et al. 2006; Testerman and Morris 2014; Shojaee Tabrizi et al. 2015). The pancreatic diseases associated with *H. pylori* infection are acute, chronic, and autoimmune pancreatitis, pancreatic cancer, and diabetes *mellitus* (DM) (Manes et al. 2003; Nilsson et al. 2006; Rieder et al. 2007; Rabelo-Gonçalves et al. 2015). The majority data about *H. pylori* and its potential participation in the development of pancreatic diseases refers to autoimmune forms of chronic pancreatitis and pancreatic adenocarcinoma. Evidence indicates a modest increased pancreatic cancer risk in infected individuals (Kunovsky et al. 2021). Systematic reviews and meta-analyses have linked *H. pylori* infection with insulin resistance, DM, neglected glycaemic control and diabetic complications, as well as other components of metabolic syndrome and non-alcoholic fatty liver disease (Polyzos et al. 2021). It has been proposed that the increase of gastrointestinal permeability, caused by *H. pylori*, contributes to systemic malfunction and the development of diabetes (De Kort et al. 2011). Moreover, Song et al. (2021) evaluated the *H. pylori* eradication rate between patients with and without DM, demonstrating an approximately double pooled odds ratios (OR) of unsuccessful eradication in the former than the latter (OR: 2.08; 95% CI: 1.56e2.77) (Song et al. 2021).

Although many animal models have been used for the analysis of *H. pylori* infection, such as mice, rats, cats, pigs, and gerbils (Honda et al. 1998; Mendoza-Elizalde et al. 2016), the bacterial DNA presence has been reported in the pancreas of cats, although the functional implications in this tissue are still unknown (Shojaee Tabrizi et al. 2015). Since gerbil is a robust, efficient, and cost-effective model that recapitulates several features of gastric inflammation and carcinogenesis in humans induced by *H. pylori*, the objective of this work was to determine the *H. pylori* manifestation in pancreas, in order to analyse its effect on this epithelial tissue. The *H. pylori* infection in gerbils, was confirmed by the bacteria presence in gastric tissue and elevated IL-8 levels at serum. This is the first report that proved the *H. pylori* occurrence in pancreas by different methodology approaches, such as urease activity, bacterial culture, and PCR (*glmM* and *cagA* genes) and immunofluorescence (α-*Hp*, α-CagA and α-OMPs antibodies) assays. In pancreas, this bacterium produced alterations in tight junction, adherens junction and desmosome structures, as observed by the redistribution of claudin-1, claudin-4, occludin, ZO-1, E-cadherin, β-catenin, desmoglein-2 and desmoplakin I/II proteins. Furthermore, the actin-cytoskeleton was also disturbed, as reported in gastric tissue. These structural modifications produced changes in the insulin and glucagon localization, suggesting that *H. pylori* infection could affect the pancreas functions.

## Materials and methods

### *H. pylori* culture

Two *H. pylori* strains were used in this work: (1) the reference strain 26695 obtained from ATCC®; and (2) a clinical isolate 279-a derived from a 12 years old male patient with gastroesophageal reflux disease and dyspepsia, obtained and characterized in the Infectious Diseases Laboratory of the Federico Gómez Children's Hospital of Mexico. Bacilli of the clinical isolate were highly flagellated and expressed CagA+, VacA s1m1, BabA 2+, and GlmM proteins. Bacteria were grown in Casman base medium (DIBICO) supplemented with 5% defibrinated sheep blood (HEMO-PROVEEDORES) and antibiotics (3 mg/ml vancomycin, 5 mg/ml trimethoprim and 2 mg/ml amphotericin B; Invitrogen™), and incubated at 37 °C with 5% CO2 and 10% humidity.

### Animal model

Male gerbils (*Meriones unguiculatus* Hsd: MON, Harlan Teklad) of 6–8 weeks age, were fasted for 8 h, then they received intragastrically 0.5 ml of 0.2 M NaHCO_3_ and 30 min later, 500 μl Brucella broth (BD BBL) were also administrated. Then, animals were divided in four groups (n = 9 animals for each): (1) control, (2) EtOH-treated, (3) *H. pylori* (*Hp*)-inoculated, and (4) EtOH-treated plus *Hp*-inoculated. (1) Control: animals were inoculated only with Brucella broth (BD BBL). (2) EtOH-treated: gerbils received 200 μl of 60% ethanol for three times every 48 h, four months later of the experiment beginning (Ahmed et al. 2013). (3) *Hp*-inoculated: animals received a bacterial suspension (6 × 10^8^ CFU/ml) of both strains resuspended in Brucella broth, for five times every 48 h. This process was performed for three more times, leaving a month of rest between each inoculation. (4) EtOH-treated plus *Hp*-inoculated: gerbils were infected as in the previous group and treated with ethanol as previously described. All animals were fed ad libitum with special food for rodents (Labdiet 5001) and sterilized water, and maintained in 12–12 h light–dark period, 23 ± 2 °C temperature and 40–60% relative humidity, during 12 months.

### Ethics statement

The Children's Hospital of Mexico Federico Gomez fulfilled the standard of the Mexican Official Norm (NOM-062-ZOO-1999). The Ethics, Biosafety and Scientific Committees at the Health Institute, as the regulatory office for the approval of research protocols involving the use of laboratory animals and, in fulfilment of the NOM, reviewed and approved all animal experiments. This work was developed under the HIM/2011/080 protocol SSA.1005 and HIM/2023/017.SSA. The study is reported in accordance with ARRIVE guidelines.

In addition, all methods were performed in accordance with the relevant guidelines and regulations.

### Tissue procurement

The animal euthanasia was carried out 12 months after infection by exsanguination under deep sedation, using ketamine/xylazine (PISA), to obtain pancreas, stomach and blood. The blood was stored at − 70 °C for later use. The pancreas and stomach were washed with PBS (137 mM NaCl, 10 mM Na_2_HPO_4_, 1.8 mM KH_2_PO_4_, 2.7 mM KCl, pH 7.4) and divided in three parts for following assays.

### Bacterial culture from tissue

The pancreatic and gastric tissues were disintegrated in PBS. Later an inoculum was taken to sow in Casman agar culture dishes, supplemented with 5% defibrinated sheep blood and antibiotics as above. Culture dishes were incubated at 37 °C with 5% CO2 and 10% humidity, to allow the bacterial growth during 7–14 days. *H. pylori*-positive colonies were morphologically identified by their shape as small, bright, and colourless, and then selected by Gram staining. Finally, the colonies were molecularly characterized by PCR assays*.*

### Bacterial urease activity in animal tissue

To evaluate the presence of bacterial urease in stomach and pancreas, tissues were disintegrated in PBS, and samples were inoculated in Christensen's medium (Mendoza-elizalde et al. 2016). Bacteria hydrolyse urea through the enzyme urease, releasing ammonia and carbon dioxide. These products alkalinize the culture medium by turning the phenol red indicator from yellow to red.

### PCR from animal tissue

To determine the presence of *H. pylori* DNA in stomach and pancreas, tissues were lysed and DNA extraction was performed using the Wizard® genomic DNA purification kit (PROMEGA), following the manufacturer's instructions. DNA amount and integrity were measured in the EPOCH microplate reader (BioTEK) and assessed by 1% agarose gel electrophoresis, respectively. The PCR assays were performed using specific oligonucleotides for the bacterial *glmM* (forward 5ʹ-AAGCTTTTAGGGGTGTTAGGGGTTT-3ʹ and reverse 5ʹ-AAGCTTACTTTCTAACACTAACGC-3ʹ) (Smith 2004) and *cagA* genes (forward 5ʹ-AATACACCAACGCCTCCAAG-3ʹ and reverse 5ʹ-TTGTTGCCGCTTTTGCTTCC-3ʹ) (Lage et al. 1995) and the PCR Master Mix polymerase kit (PROMEGA), in a thermocycler T100 Thermal-Cycler (BioRad). PCR products were separated in a 1% agarose gel and stained with Midori Green Direct (NIPPON Genetics EUROPE). Bands were visualized in an iBright FL 1500 imaging system (Invitrogen). DNA from both *H. pylori* strains (26,695 and 279-a) were used as positive controls. In negative controls the same PCR mix and oligonucleotides were used, but without DNA template.

### Histological technique and hematoxylin–eosin (H&E) staining

Stomach and pancreas samples were fixed in 4% paraformaldehyde, kept at 4 °C, and processed for conventional embedding paraffin. Then, 6 μm thick sections were stained with haematoxylin–eosin (H&E) (Velazquez-Guadarrama et al. 2007).

### Indirect immunofluorescence

Pancreas and stomach tissues were cryoprotected with 2-methyl butane and mounted on Tissue-Tek® stand (VWR) for freezing with liquid nitrogen. Frozen samples were cut on a cryostat in 6–10 µm thick sections and placed on gelatine-coated slides. Slides were washed three times with PBS, fixed with acetone for 5 min at − 20 °C, and washed again with PBS. Then, slides were blocked for 30 min at room temperature (RT) with 0.5% IgG-free bovine serum albumin (BSA; US Biological). Later, they were incubated overnight (ON) at 4 °C with the following primary antibodies: rabbit α-*H. pylori* (α-*Hp*; 1:2,000; GeneTex), mouse α-CagA (1:50; Santa Cruz Biotechnology), mouse α-OMPs from *H. pylori* (1:100; Santa Cruz Biotechnology), rabbit α-claudin-1 (1:50; Invitrogen), mouse α-claudin-4 (1:40; Life Technologies), mouse α-occludin (1:100; Invitrogen), rabbit α-ZO-1 (1:50; Invitrogen), rat α-E-cadherin (1:500; Santa Cruz Biotechnology), mouse α-β-catenin (1:300; Santa Cruz Biotechnology), mouse α-desmoglein-2 (1:30; Santa Cruz Biotechnology), rabbit α-desmoplakin I/II (1:40; Santa Cruz Biotechnology), rabbit α-insulin (1:250; Novus Biologicals), and rabbit α-glucagon (1:200; Biogenex Laboratories). Next day, samples were washed three times with PBS and incubated with fluorochrome-coupled species-specific secondary antibodies such as: α-mouse Alexa 488 (1:100; Invitrogen) or α-rabbit Alexa-647 (1:100; Invitrogen). Some samples were additionally incubated with phalloidin-FITC (1:400; Invitrogen). Nuclei were stained with 2.5 µg/ml 4ʹ,6-diamino-2-phenylindole (DAPI). Sections were examined through a Carl Zeiss LMS 700 confocal microscope and processed with ZEN 2009 Light Edition Software (Zeiss). ImageJ 1.52q software was employed to evaluate the net fluorescence intensity per pixel of immunodetected proteins in 20 confocal images (laser sections: 0.5 μm in *xy*-planes) obtained from four grid squares and randomly selected.

### IL-8 measuring

From the sera previously obtained, the IL-8 measurement was carried out with the help of the IL-8/cxcl15 ELISA kit (MyBioSuource), following the manufacturer’s instructions. 96 well plates coated with 100 μl of IL-8 standards or test samples (diluted at 1/4 with sample dilution buffer) were incubated at 37 °C for 90 min. Control (blank) wells contained only sample dilution buffer. Plates were washed twice with washing buffer and incubated for 60 min at 37 °C with 100 μl biotin-labelled antibody (1:100). After three washes, 100 μl of HRP-streptavidin conjugate (1:100) was added and plates were incubated 30 min at 37 °C. Five more washes were carried out and 90 μl of TMB substrate were added into the wells, incubating in dark 10–20 min at 37 °C. The stop solution (50 μl) was added, and immediately the plates were measured at λ = 450 nm in the EPOCH microplate reader (BioTEK). All experiments were performed by duplicate.

### Statistical analysis

Results displayed in this work represent the mean and standard error. Statistical analyses were performed by Chi-square for trend, ANOVA and t-Student tests, using GraphPad Prism 6.0 software. Statistically significant differences are designated with asterisks in the table and graphs (∗p < 0.05, ∗∗p < 0.01 or ***p < 0.001).

## Results

### *H. pylori* is present in the pancreas, but does not produce evident morphological changes

**Fig. 1:**
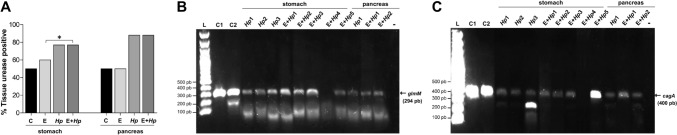
Presence of bacterial genes at pancreas and percentage of gerbils infected with *H. pylori*. **A** Urease test in homogenized pancreas and stomach. Data from groups *Hp*-inoculated and Et-OH treated + *Hp*-inoculated were statistically compared with Et-OH treated animals, by Chi-square test for trend using the GraphPad Prism software. *p < 0.05. **B, C** PCR assays of *glmM* (**B**) or *cagA* (**C**) genes, using DNA from bacterial cultures derived from pancreas and stomach tissues as templates. Bacterial DNA from 26,695 and 279-a strains were used as positive controls (C1 and C2). Negative control (−): DNA template was omitted. L: DNA ladder. C: control; E: EtOH-treated; *Hp*: *H. pylori*-inoculated; E + *Hp*: EtOH-treated plus *Hp*-inoculated. n = 9

The effect of *H. pylori* on the gastric epithelium has been widely studied, evidencing clear disturbance of the barrier integrity. The opening of tight junctions and erosion of gastric epithelium could allow the bacterial entrance to blood stream and other tissues (Alzahrani et al. 2014). Recent findings seem to correlate the *H. pylori* seropositivity with pancreatic diseases (Bulajic et al. 2014); however, there are scarce studies indicating the presence of bacterial molecules in pancreas, and even less evidence exists for the presence of the complete bacteria in this tissue. Here, we developed a rodent model of infection based on gerbils, and to promote the *H. pylori* invasion to other tissues, we treated experimentation animals with ethanol in order to produce ulcers, as previously demonstrated (Ahmed et al. 2013). Thus, four animal groups were employed: (1) control, (2) EtOH-treated, (3) *Hp*-inoculated, and (4) EtOH-treated plus *Hp*-inoculated; and experiments were performed after 12 months of treatment. Firstly, we evaluated whether animals were successfully infected by *H. pylori* through the urease test, inoculating a portion of gastric or pancreatic tissue in urea-agar for bacterial culture. Gastric tissue of animals from groups 3 and 4 presented around 77% positivity to the urease test (Fig. 1A). Of note, animals not inoculated also presented positivity for this test. Nevertheless, the percentages of urease activity in groups 3 and 4 are higher, in comparison to the control and EtOH-treated animals (50 and 60% urease-positive, respectively). In pancreatic tissue, groups 3 and 4 were 90% positive to the urease test, relative to groups 1 and 2, which exhibited 50% of positivity (Fig. 1A).

To confirm the infection, a portion of gastric and pancreatic tissues was inoculated in Casman agar supplemented with sheep's blood and selective antibiotics (vancomycin, trimethoprim, and amphotericin B) (Mendoza-Elizalde et al. 2016) for *H. pylori* growth. Then, an alike *H. pylori* colony (small, bright, and colourless) was reseeded and morphologically characterized by Gram staining. The colonies with similar morphology from originally inoculated strains (26,695 and 279-a), came from the stomachs of 3 and 4 animals and from the pancreas of 1 and 2 gerbils, of groups 3 and 4, respectively. Later, genomic DNA derived only from these bacterial colonies was obtained. PCR assays were performed and representative genes from *H. pylori*, as *glmM* (Espinoza et al. 2011) (encoding for a phosphoglucosamine mutase) and *cagA* (encoding for the CagA virulence factor) (Jeyamani et al. 2018) were detected. By using this DNA as template, and specific oligonucleotides, amplicons of *glmM* (240 bp) and *cagA* (400 bp) genes were revealed only in groups 3 and 4, but not in control and EtOH-treated animals (Fig. 1B, C). Of note, both genes were present in pancreas of *Hp*-inoculated animals. DNA derived from bacterial lysates from strains 26,695 and 279-a were used as template in positive controls. The results obtained from urease test, bacterial cultures only with *Hp*-features and PCR assays were concentrated in Table 1.

**Table 1:** Number of gerbils positive to urease test, *Hp* culture, and *glmM* and *cagA* gene amplification by PCR assays among different groups

Stomach				
Group	Urease test	*Hp* culture	PCR assay	
			*glmM* gene	*cagA* gene
Control (n = 4)	2	0	0	0
Et-OH treated (n = 5)	3	0	0	0
*Hp*-inoculated (n = 9)	7 (ns)	3 (ns)	3 (ns)	3 (ns)
Et-OH treated + *Hp*-inoculated (n = 9)	7 (ns)	5 (*)	4 (*)	4 (*)
*Pancreas*				
Control (n = 4)	2	0	0	0
Et-OH treated (n = 4)	2	0	0	0
*Hp*-inoculated (n = 9)	8 (ns)	1 (ns)	1 (ns)	1 (ns)
Et-OH treated + *Hp*-inoculated (n = 9)	8 (ns)	2 (ns)	2 (ns)	2 (ns)

Data from groups *Hp*-inoculated and Et-OH treated + *Hp*-inoculated were statistically compared with Et-OH treated animals, by Chi-square test for trend using the GraphPad Prism software

*ns* non-significant

*p < 0.05

Importantly, not all *Hp*-inoculated animals from groups 3 and 4 were infected, thus, we considered as animals infected, those presenting the *glmM* and *cagA* genes in the gastric tissue. In addition, in the pancreas of some of these animals, these genes were also amplified. Then, 3/9 and 4/9 gerbils from groups 3 and 4, respectively, were infected by *H. pylori*. In following experiments, only the infected animals were employed (at least 3 animals per group).

**Fig. 2:**
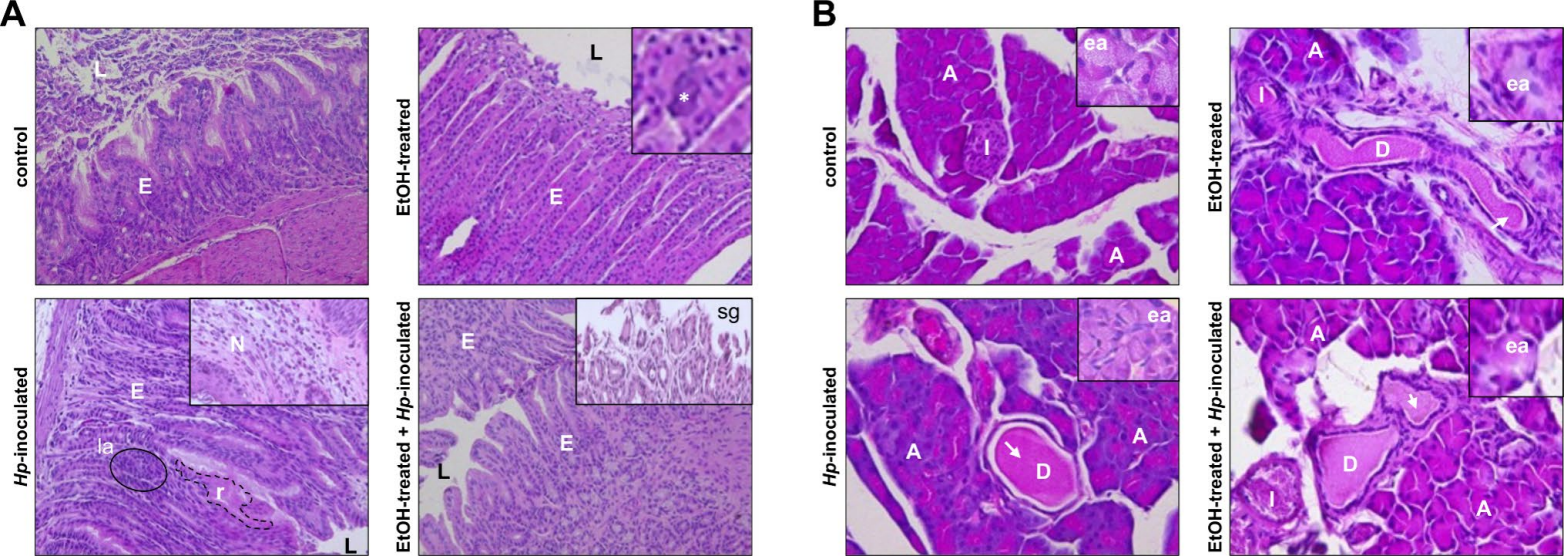
Histopathology of stomach and pancreas tissues from gerbils. **A, B** Sections of gerbil’s stomach (**A**) and pancreas (**B**) tissues were processed for H&E staining and observed under the light microscope. Representative images from one animal are shown for each group (n = 9). Magnification 40X. L: lumen; E: epithelium; N: neutrophils; la: lymphoid accumulation; r: regeneration; sg: superficial gastritis; A: acinus; I: Langerhans islet; D: duct; ea: empty acini. Asterisk: calcifications. Arrow: ductal plugging

Next, we analysed the morphological damage produced in gastric and pancreatic tissues by *H. pylori*, through haematoxylin–eosin staining. In the control and EtOH-treated groups, no significative changes were found in both studied tissues. In stomach of animals from group 2, only few calcifications were detected (Fig. 2A). In pancreas, some ductal plugging and empty zymogen granules in the acini were observed, similar to those found in control animals (Fig. 2B). In the *Hp*-inoculated animals, the gastric sections revealed lymphoid accumulation, tissue regeneration and presence of neutrophils (Fig. 2A). Meanwhile, the pancreas images were similar to control and EtOH-treated groups (Fig. 2B). The gastric preparations from EtOH-treated plus *Hp*-inoculated animals (group 4) showed atrophy, tissue regeneration and superficial gastritis (Fig. 2A); while pancreatic samples look similar to other groups and no alterations were detected (Fig. 2B). All these alterations at the stomach and pancreas were exhibited only by infected animals from the *Hp*-inoculated groups.

These findings suggest that *H. pylori* reaches the pancreas and maintains its proliferation ability, as exhibited when infected pancreatic tissue was homogenized and bacteria was cultured in agar medium, although evident morphological changes were not revealed.

### *Helicobacter pyloribacteria* and some of their proteins are present in pancreas

**Fig. 3:**
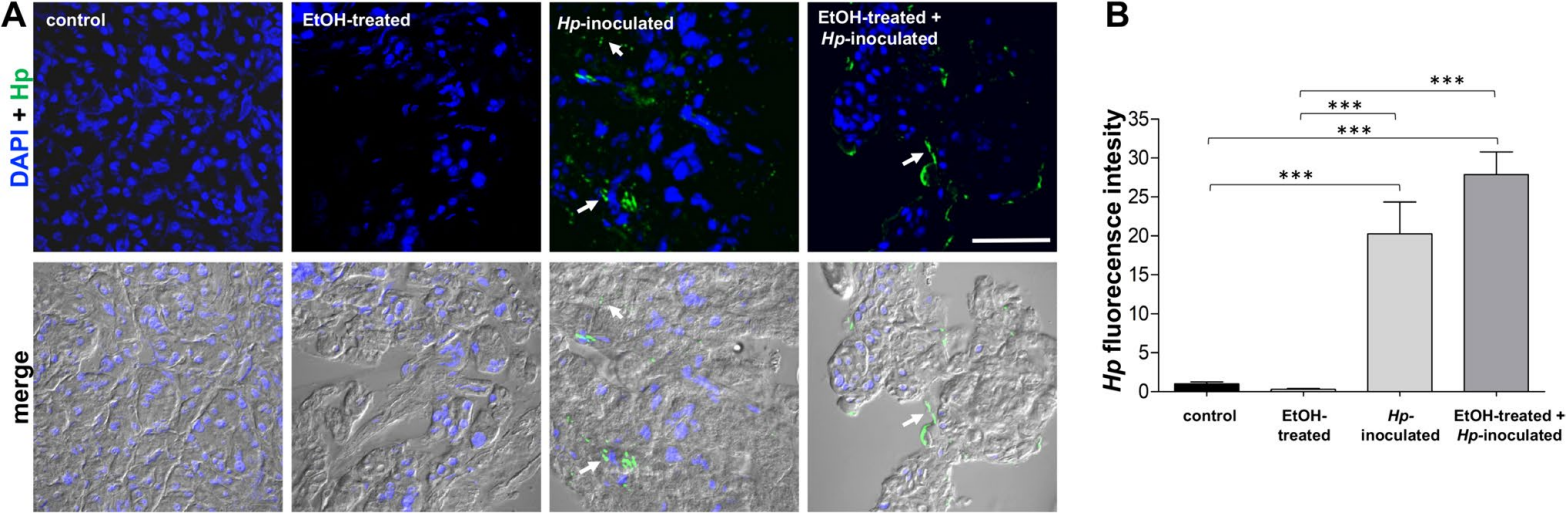
Presence of *H. pylori* at gerbil pancreas.** A** Pancreas sections were processed for immunofluorescence assays using ⍺-*Hp* antibody (green). Nuclei (blue) were stained with DAPI. Merge: fluorescence images overlapped with the phase-contrast bright field. Arrows: *Hp* localization outside the cells. Bar = 50 μm. Representative images from one animal are shown for each group (n = 3). **B** Green fluorescence intensity was measured by pixels using the ImageJ software. (***)p < 0.001. All groups were statistically compared regarding control and EtOH-treated animals

To confirm the presence of the *H. pylori* bacteria in pancreas, immunofluorescence (IF) assays were performed using an anti-*Hp* antibody. The bacteria were only observed outside of the cells in animals of groups 3 and 4, and no specific fluorescence signal was detected in control and EtOH-treated gerbils (Fig. 3A). The fluorescence intensity was quantified, and it resulted elevated in *Hp*-inoculated animals, and even higher in group 4, suggesting that the ulcers produced by EtOH promoted the bacterial access beyond the gastric epithelium (Fig. 3B).

**Fig. 4:**
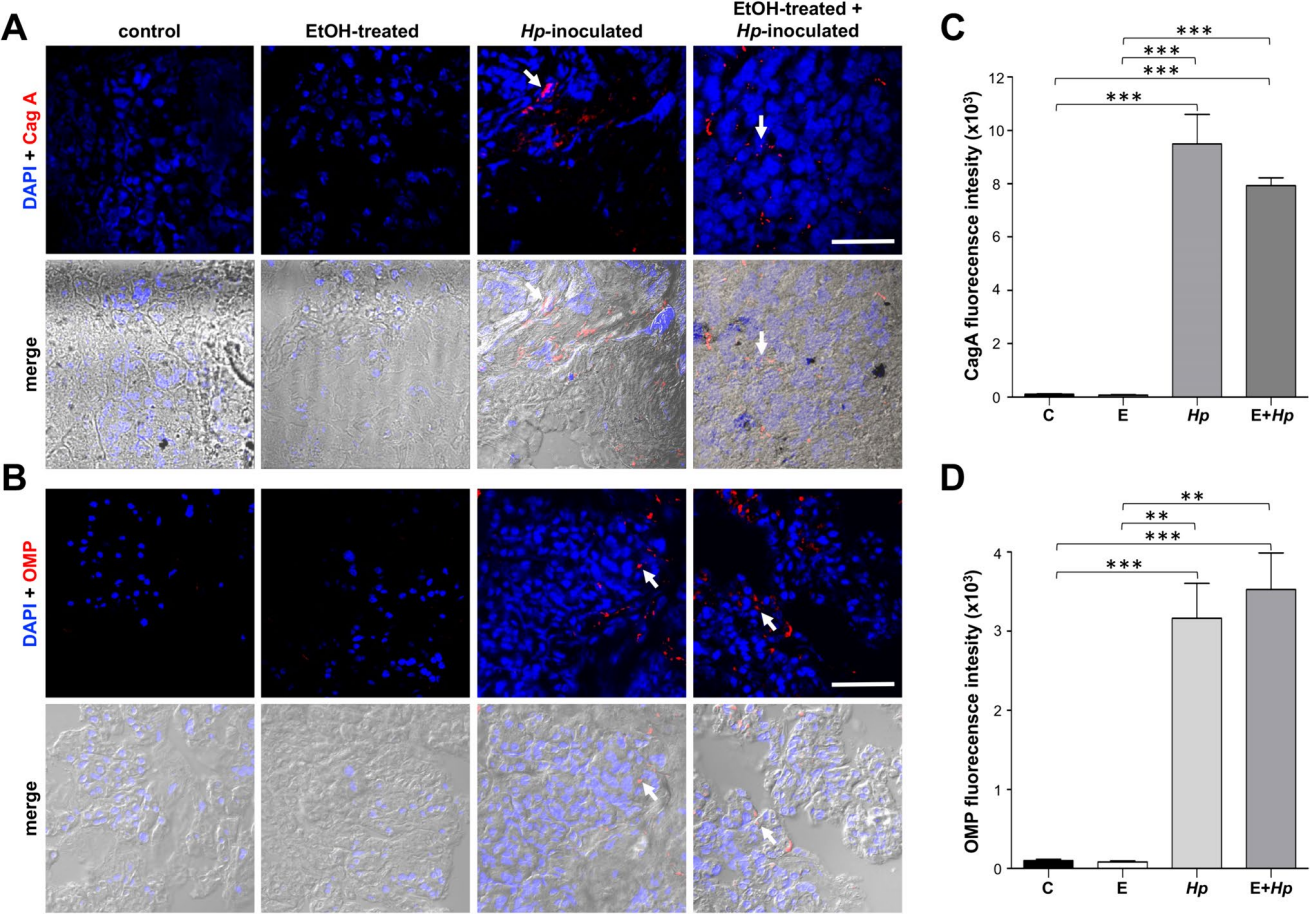
Presence of *H. pylori* proteins at gerbil pancreas. **A, B** Pancreas sections were processed for immunofluorescence assays using ⍺-CagA (**A**) or ⍺-OMPs (**B**) antibodies (red). Nuclei (blue) were stained with DAPI. Arrows: localization of bacterial proteins inside the cells. Merge: fluorescence images overlapped with the phase-contrast bright field. Bar = 50 μm. Representative images from one animal are shown for each group (n = 3). **C, D** Red fluorescence intensity of CagA (**C**) or OMPs (**D**) was measured by pixels using the ImageJ software. All groups were statistically compared regarding control and EtOH-treated animals. (**)p < 0.01 and (***)p < 0.001. C: control; E: EtOH-treated; *Hp*: *Hp*-inoculated; E + *Hp*: EtOH-treated plus *Hp*-inoculated

In addition, we also investigated whether some bacterial proteins were localized in pancreas. By IF experiments, CagA (Fig. 4A) and OMPs (Fig. 4B) proteins from *H. pylori* were mainly detected inside of the pancreatic cells of *Hp*-inoculated animals from groups 3 and 4. In control and EtOH-treated gerbils, no specific fluorescence signal was observed (Fig. 4A, B). The quantification of fluorescence intensity confirmed the presence of both bacterial proteins only in *Hp*-infected animals (Fig. 4C, D). As control, the gastric epithelium was analysed by IF assays for the localization of *H. pylori* and CagA and OMP proteins, and accordingly, only infected animals showed signals (Figs. S1 and S2).

These results corroborate that *H. pylori* is able to reach the pancreas and produce there some virulence factors, which are in some way internalized.

### *Helicobacter pylori* disturbs intercellular junction proteins in pancreas

**Fig. 5:**
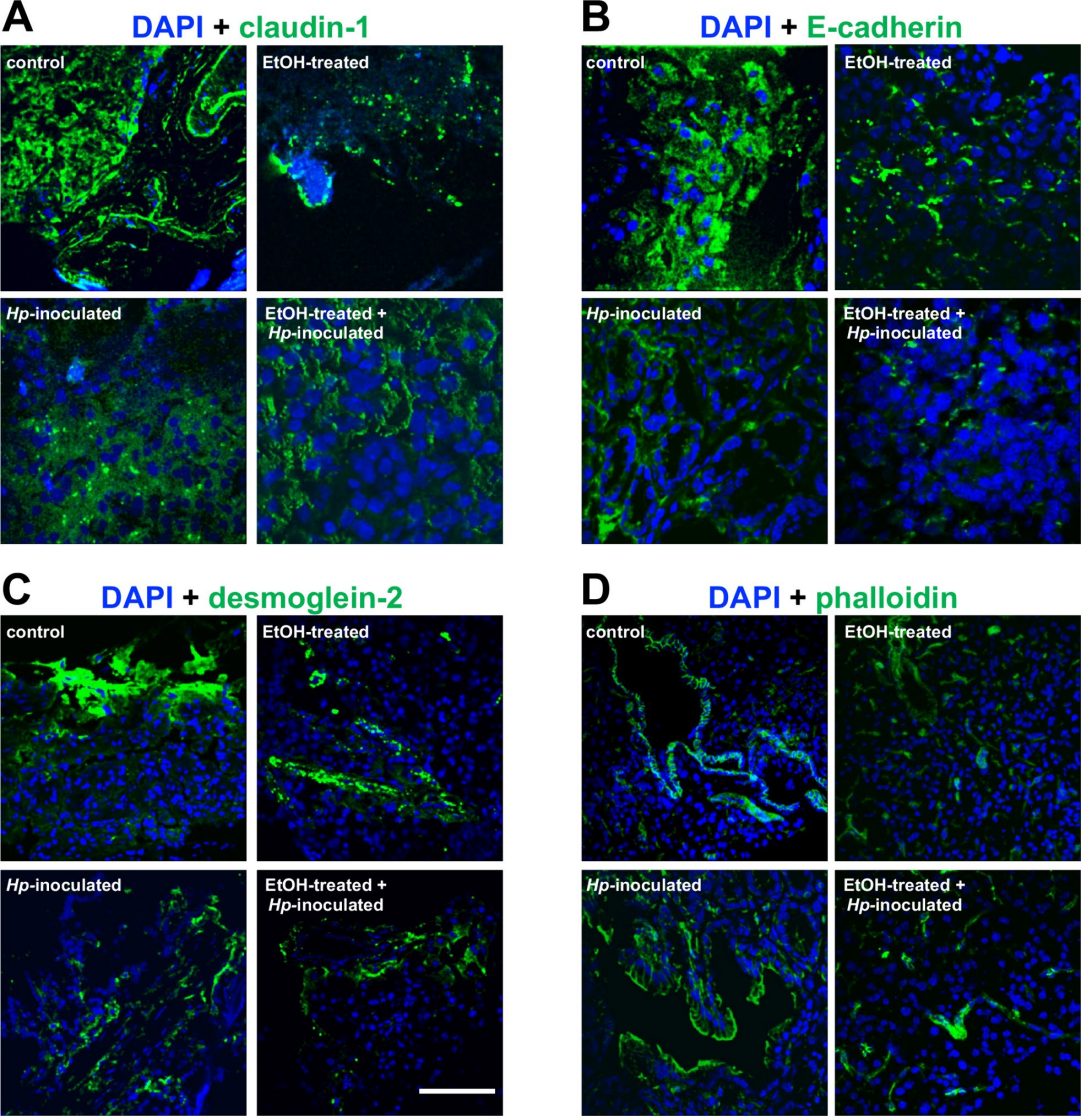
Alterations of intercellular junction proteins at gerbil pancreas by *H. pylori* infection. **A–C** Pancreas sections were processed for immunofluorescence assays using ⍺-claudin-1 (**A**), ⍺-E-cadherin (**B**) or ⍺-desmoglein-2 (**C**) antibodies (green). **D** Polymerized actin was stained with phalloidin-FITC (green). Nuclei (blue) were stained with DAPI. Bar = 50 μm. Representative images from one animal are shown for each group (n = 3)

**Fig. 6:**
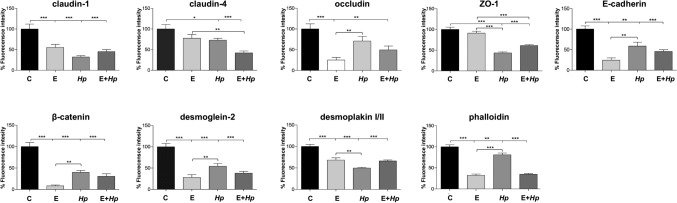
Levels of intercellular junction proteins at gerbil pancreas by *H. pylori* infection. Fluorescence intensity of occludin, claudin-1, claudin-4, ZO-1, E-cadherin, β-catenin, desmoglein-2, desmoplakin I/II and phalloidin was measured by pixels using the ImageJ software. All groups were statistically compared regarding control and EtOH-treated animals. (*)p < 0.05, (**)p < 0.01 and (***)p < 0.001. C: control; E: EtOH-treated; *Hp: Hp*-inoculated; E + *Hp*: EtOH-treated plus *Hp*-inoculated. n = 3

The functional state of pancreas is regulated by several adhesion structures, including tight and adherens junctions and desmosomes (Jimenez-Caliani et al. 2017; Myo Min et al. 2022), which are essential for homeostasis, and to preserve the normal epithelial structure and regulate secretion processes of this tissue (Sato et al. 2019). Preserving the normal function of junctional molecules and avoiding their abnormal distribution, the pancreas structure is maintained, and the development of diseases is prevented. Therefore, we studied how the *H. pylori* infection affects the distribution of some adhesion molecules. By IF assays and using specific antibodies, we analysed the localization changes in tight junction proteins as claudin-1, claudin-4, occludin, and ZO-1; in adherens junction molecules as E-cadherin and β-catenin; and in desmosomes proteins as desmoglein-2 and desmoplakin I/II. The localization of claudin-1, mainly distributed in the cellular borders in control animals, changed in EtOH-treated gerbils, diminishing particularly from plasma membrane (Fig. 5A). While, in the *Hp*-inoculated group, this protein was internalized toward the cytoplasm and disappeared from cellular borders. In animals infected with *H. pylori* and EtOH-treated, a dramatic reduction of claudin-1 was observed (Fig. 5A). Similar results were displayed for claudin-4, and a decreasing trend was observed for occludin and ZO-1 (Fig. S3). The fluorescence intensity was quantified and significant differences among treatments were displayed (Fig. 6). In gastric tissue, a comparable redistribution of these proteins was observed, having the group 4 the more dramatic effect (Figs. S4–S6). Delocalization and decrease of E-cadherin and desmoglein-2 were observed in pancreas from groups 2, 3 and 4, with respect to the control animals (Fig. 5B, C). Results were confirmed by fluorescence quantification, and a similar behaviour was revealed for β-catenin and desmoplakin I/II (Figs. 6 and S3). In stomach, analogous patterns were detected (Figs. S4–S6).

Dynamic rearrangements of the actin cytoskeleton constitute a hallmark of *H. pylori* infected gastric cells that conducts to invasive growth toward other tissues. Therefore, we analysed the actin distribution in pancreas, observing a main localization at cellular border of endothelial cells and rounding acini in control animals (Fig. 5D). In treated gerbils (groups 2–4), an important reduction was detected when actin-fluorescence intensity was quantified (Fig. 6). It suggests that as in stomach (Figs. S4 and S6), this bacterium is also producing actin cytoskeleton remodelling in pancreas, which probably affects the intercellular junctions and functions of this organ, such as disturbing the hormone secretion.

### *H. pylori* infection alters hormones distribution at pancreas

**Fig. 7:**
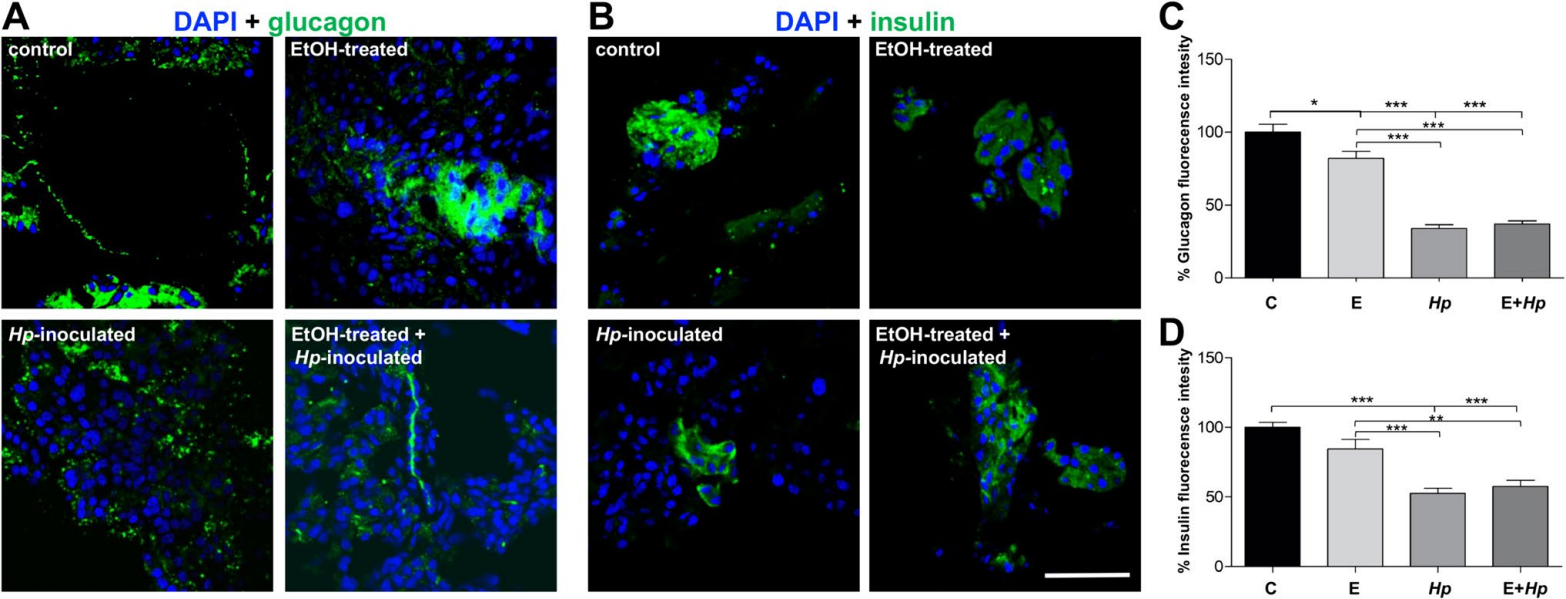
Modifications of endocrine pancreas of gerbils infected with *H. pylori*. **A, B** Pancreas sections were processed for immunofluorescence assays using ⍺-glucagon (**A**) or ⍺-insulin (**B**) antibodies (green). Nuclei (blue) were stained with DAPI. Bar = 50 μm. Representative images from one animal are shown for each group (n = 3). **C, D** Fluorescence intensity of glucagon (**C**) and insulin (**D**) was measured by pixels using the ImageJ software. All groups were statistically compared regarding control and EtOH-treated animals. (**)p < 0.01 and (***)p < 0.001. C: control; E: EtOH-treated; *Hp*: *Hp-*inoculated; E + *Hp*: EtOH-treated plus *Hp-*inoculated

The endocrine function of pancreas is to regulate the blood glucose levels through the secretion of two main hormones, insulin and glucagon (Röder et al. 2016). In this work, we studied the effect of *H. pylori* infection on the distribution of both hormones. The EtOH-treatment produced a significant re-localization of glucagon toward the cytoplasm, whereas the infection in groups 3 and 4, significantly diminished the amount of this protein in pancreatic tissue (Fig. 7A, C). Insulin was also reduced in pancreas by the infection of *H. pylori,* and only a redistribution was detected by the treatment with ethanol (Fig. 7B, D).

### IL-8 levels are increased in gerbils infected with *H. pylori*

**Fig. 8:**
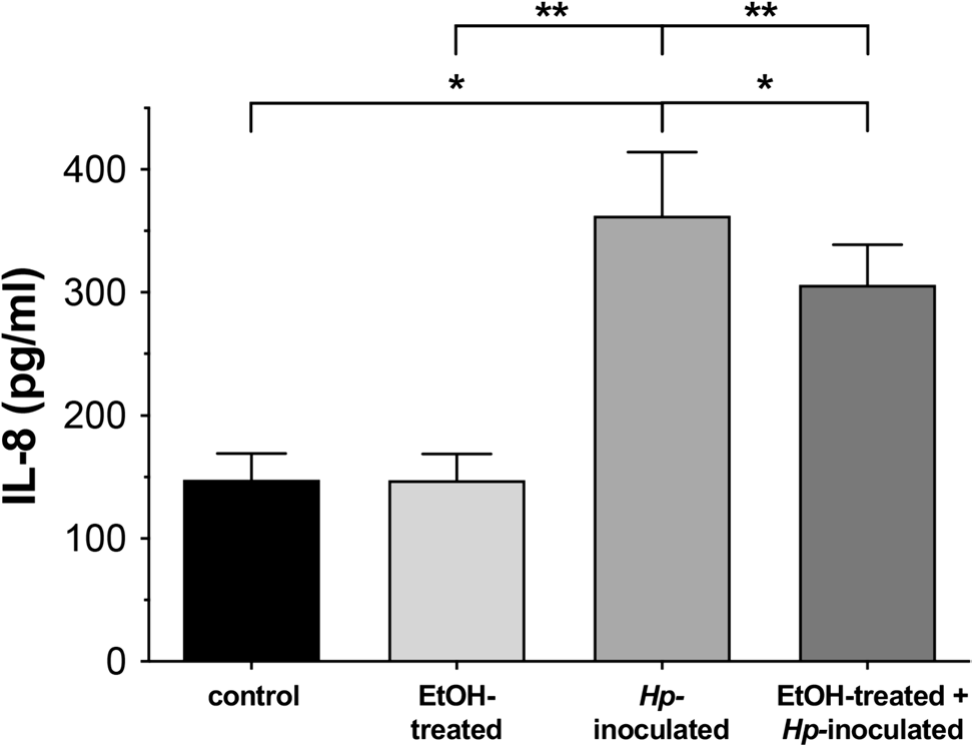
Serum levels of IL-8 in gerbils infected with *H. pylori*. Measuring of IL-8 in serum of Mongolian gerbils by ELISA. All groups were statistically compared regarding control and EtOH-treated animals. (*)p < 0.05 and (**)p < 0.01. n = 3

Persistent *H. pylori* infection confers an increased risk for gastric diseases involving various cytokines modifications related to the inflammatory immune response. The most characterized pro-inflammatory cytokine production by this bacterial infection is IL-8. Here, we evaluated IL-8 levels in sera from different gerbil groups. In control and ulcerated animals, the cytokine concentration remained unchanged; but in *Hp*-infected gerbils (groups 3 and 4) we observed a significant increased level (more than twice regarding the control group) of IL-8 (Fig. 8). These findings suggest that *H. pylori* infection promotes a mechanism for the heightened inflammatory response, as reported in human and other animal models (Sharma et al. 1998; Zhao et al. 2015).

In general, our data indicate that gerbils are susceptible to *H. pylori* infection and, in some cases, it was promoted by ulcers production. This bacterium penetrates the stomach and can reaches the pancreas, where it internalizes some virulence molecules such as CagA and OMPs. Even when no evident morphological damage was produced in this organ, *H. pylori* is able to impair the intercellular junction proteins from the pancreatic epithelium, possibly affecting the endocrine function, as suggested by alterations in glucagon and insulin distribution in infected animals. The tissue damage in gastric and pancreas was accompanied by an inflammatory systemic response, as confirmed by the increase of IL-8 levels due to *H. pylori* infection.

## Discussion

Most of studies related to *H. pylori* to date, are focused in the gastric epithelium injury produced by this bacterium, nevertheless, its extragastric presence is gaining relevance recently, due to the relationship with several diseases in distinct organs and tissues (Gravina et al. 2020; Kunovsky et al. 2021). Particularly, in pancreas, *H. pylori* infection has been associated to pancreatitis, diabetes and cancer (Rabelo-Gonçalves et al. 2015). Nevertheless, the mechanisms to achieve this organ and the effect in the pancreatic epithelium have not been elucidated yet. Therefore, in this study, we used an infection model in gerbils to probe the presence of *H. pylori* in the pancreas. There, the bacterial infection altered proteins of the intercellular junctions and the localization of some hormones, and increased levels of IL-8 in serum.

*H. pylori* infects humans, but several animal models are currently used to elucidate the mechanisms of infection. However, in experimentation models it is difficult to obtain efficient rates of infection, due to the complex gastric epithelium response that, just in human, could take up to decades to develop symptoms and gastric diseases. Recently, gerbils have been used as models for the study of this infection, since they accurately resemble gastric inflammation and carcinogenesis produced by *H. pylori* in humans; besides, it is a competent, strong, and low-cost rodent model (Ansari and Yamaoka 2022). In Mongolian gerbils, *H. pylori* colonizes the gastric mucosa, producing inflammatory infiltrates in the lamina propria with the presence of neutrophils and mononuclear leukocytes (Di et al. 2020); however, alterations in other organs have not been studied yet. In pancreas of cats, *H. pylori* has been detected by PCR (Shojaee Tabrizi et al. 2015) and in human, this bacterium has been related with pancreatitis and pancreatic cancer (Manes et al. 2003; Rieder et al. 2007). The pathogenesis and evolution of idiopathic forms of pancreatitis, is associated with changes in the secretion of the exocrine pancreas due to *H. pylori* infection. Furthermore, the altered epithelial gastric barrier allows the colonization of other pathogens, promoting that pancreatitis becomes chronic, and eventually, contributing to cancer development (Nilsson et al. 2006; Rieder et al. 2007; Dore et al. 2008; Bulajic et al. 2014; Rabelo-Gonçalves et al. 2015; Kunovsky et al. 2021).

Other species of *Helicobacter*, such as *Helicobacter hepaticus*, cause chronic hepatitis and hepatocellular carcinoma (HCC) in mice; and *Helicobacter* spp. have been identified in the liver of patients with cholestatic diseases and in HCC derived from non-cirrhotic liver (Rocha et al. 2005). According to some theories, *Helicobacter* spp. could go from the stomach to the liver via the duodenum and biliary tract or could enter the liver from the bloodstream via the hepatic portal vein (Pellicano et al. 2008).

In this work, we generated a gerbil model for *H. pylori* infection during 12 months; in addition, to promote the bacterial access to the organism, ulcers were produced by EtOH-treatment. This treatment has already been employed in other rodent models as rats, causing ulcers successfully, as observed by H&E staining (Ahmed et al. 2013). The presence of *H. pylori* in stomach and pancreas was demonstrated here by urease test, bacterial cultures derived from these tissues and PCR assays. *Hp*-inoculated animals presented more urease activity than control and EtOH-treated gerbils. In stomach, the enzymatic activity present in the latter groups could be due to the enzymes expressed by proteobacteria of the genus *Proteus* spp. or others (Heimesaat et al. 2014). Whilst, in pancreas, the positivity of the test is partially produced by the components of this tissue, such as digestive enzymes and sodium bicarbonate, which eventually protects the duodenum by neutralizing the acid that comes from the stomach (Pandol 2017). These components make the urea-agar mildly alkaline, hence, giving a positive result. Therefore, the urease test is not adequate to determine with certainty the *H. pylori* infection. Then, to specifically determine the presence of *H. pylori* at pancreas, bacterial cultures were performed by using tissues from stomach and pancreas, and employing antibiotics (vancomycin, trimethoprim and amphotericin B) (Mendoza-Elizalde et al. 2016) for a selective *H. pylori* growth. Only colonies with similar morphology to initially inoculated strains were selected and came from stomach (3 and 4 animals) and pancreas (1 and 2 gerbils) of groups 3 and 4, respectively. From these colonies, bacterial DNA was isolated, and the *glmM* and *cagA* genes were PCR-amplified. Hence, the rate of infection was 33 and 44% for groups 3 (*Hp*-inoculated) and 4 (*Hp*-inoculated + EtOH-treatment), respectively. Albeit the EtOH-treatment favoured the bacterial infection, the percentage of infection obtained was still lower than those obtained in previous studies (Fig. 1) (Velazquez-Guadarrama et al. 2007; Cortés-Márquez et al. 2018). The pronounced influence of genetic diversity, particularly, regarding to the immune system in the infection models, could explain the differential response to the bacterial infection. Therefore, it seems necessary to increase the time of infection until 18 months and the number of animals per group.

In order to corroborate if *H. pylori* or some of its virulence factors reach the pancreas, we used specific antibodies against *H. pylori*, CagA and OMP's. By immunofluorescence experiments, signals for *H. pylori*, CagA and OMPs were only detected in infected groups (3 and 4); while in groups 1 and 2, no fluorescence was distinguished. These findings suggest that *H. pylori* and some bacterial proteins are achieving the pancreas, displaying a pattern localization similar to that described in an infected stomach (Fig. 4). Alternatively, these proteins could arrive to the pancreas through outer membrane vesicles (OMVs) derived from bacteria or by exosomes produced from infected gastric cells (Chen et al. 2018; Jarzab et al. 2020).

Morphologically, infected animals showed lesions in the gastric epithelium, such as regeneration, lymphoid accumulation, and superficial gastritis, that could be attributed to the presence of *H. pylori*. In other studies, during prolonged infections, severe inflammation results in the loss of parietal and chief cells, usually accompanied by hyperplasia of the mucous cells of the neck (Ohkusa et al. 2003; Ansari and Yamaoka 2022). Nevertheless, in our model, no apparent morphological injuries were observed in the pancreas of infected gerbils. In all groups, empty acini and plugging in some ducts were detected, as reported in the pancreas of normal and diabetic gerbils (Fig. 2) (Li et al. 2016).

Taking into account that histologically there were no changes in the pancreatic tissue, we reviewed the epithelial structure by analysing the localization of proteins from the intercellular junctions. Proteins from the tight junction (claudin-1, claudin-4, occludin and ZO-1), adherens junction (E-cadherin and β-catenin) and desmosomes (desmoglein-2 and desmoplakin I/II) were delocalized from the cellular borders towards the cytoplasm, mainly in the groups 3 and 4, with respect to group 1. These localization changes correlated with a reduction in the amount of these proteins, as revealed by fluorescence quantification, suggesting that the expression of intercellular molecules is affected by *H. pylori* infection, as occurs in the stomach (Costa et al. 2013) (Fig. 6).

In the gastric mucosa the OMPs expression helps *H. pylori* to attach to gastric epithelial cells at the primary stage of infection and rises the virulence of this bacterium. OMPs also cooperate with other virulence factors such as CagA and VacA to increase the release of inflammatory factors, neutrophil infiltration, and helping to the colonization, persistent infection, and severe clinical consequences (Xu et al. 2020). Besides, *H. pylori* internalizes CagA through its T4SS system, which can be also secreted together with other virulence factors. In the epithelial cells, this oncoprotein induces the delocalization of intercellular junction proteins such as ZO-1, E-cadherin, and β-catenin, which leads to the epithelial barrier impairment (Alzahrani et al. 2014). In epithelial cells monolayers like MDCK, CagA causes polarity defects characterized by alterations of ZO-1 and E-cadherin, in a PAR1b-depending manner (Takahashi-Kanemitsu et al. 2020). Moreover, CagA has the ability to physically interact with the cytoplasmic domain of E-cadherin. The CagA/E-cadherin interaction interferes with and destabilizes the formation of the E-cadherin/ β-catenin complex and abnormally re-localizes the membrane-bound portion of β-catenin to the nucleus, where it activates Wnt-target genes in a β-catenin/TCF-dependent manner (Takahashi-Kanemitsu et al. 2020). Otherwise, the *H. pylori* serine protease HtrA, opens cell-to-cell junctions through cleavage of occludin, claudin-8, and E-cadherin, thus inducing the disintegration of their epithelial barrier functions (Tegtmeyer et al. 2017). In gastric epithelial cells, such as NCI-N87 and MKN28, HtrA also cuts the desmosomal cadherin, desmoglein-2. Hence, tight junctions, adherent junctions, and desmosomes are targets of this serine protease (Bernegger et al. 2021). Thus, the HtrA activity is necessary for paracellular transmigration of *H. pylori* across polarized cell monolayers to reach basolateral membranes and the CagA translocation across ɑ5β1 integrin (Tegtmeyer et al. 2017).

In this context, it is possible that *H. pylori* pathogenicity factors such as urease, OMPs, CagA, VacA and HtrA, could induce host cell signalling involved in altering cell-to-cell permeability, to impair the gastric epithelial barrier and then, the bacteria or these factors can be internalized and reach deeper tissues. In pancreas, the effect of these virulence factors over junctions is similar to that described in stomach, as we observed in this work. The breakdown of the pancreatic ductal barrier is known to contribute to the pathophysiology of pancreatitis and the development of pancreatic cancer because tight junctions in the pancreas are crucial regulators of physiologic secretion (Rieder et al. 2007; Kojima et al. 2013). Various inflammatory mediators and carcinogens can trigger tight junction disassembly and disruption of the pancreatic barrier, however, signalling events involved remain poorly understood. Furthermore, in pancreas, the adhesion molecules are crucial for proliferation, cell migration, and signal transduction, as well as in the development and tissue repair. When the cell–cell adhesion between endothelium and/or pancreatic acinar cells weakens, the accompanying interstitial oedema encourages the migration of inflammatory cells and disturbs the integrity of the tissue. In pancreatitis, occludin, claudin-1 and ZO-1 are decreased, but no changes in claudin-4 have been reported; whereas E-cadherin and β-catenin are dissociated from the plasma membrane and condensed in the cytosol of acinar cells (Sato et al. 2019). Moreover, E-cadherin is important for maintaining the architecture and homeostasis of the exocrine part and its absence contributes in the development of pathogenic conditions, such as pancreatitis or pancreatic cancer (Serrill et al. 2018). In the pancreas of the mouse model, the loss of desmoplakin expression resulted in the disruption of desmosomal adhesions, that can promote increased local tumour invasion, independently of the adherens junction status (Chun and Hanahan 2010). Altogether, these findings could explain why in this work we observed that *H. pylori* infection produced changes in all these proteins, which were re-localized from the plasma membrane towards the cytoplasm, and also significantly diminished (Fig. 6).

Epithelial structure is also maintained by the cytoskeleton and dynamic rearrangements of actin; but in *H. pylori* infected gastric epithelial cells, important changes in actin lead to the development of aberrant morphological changes, cell migration and invasive growth (Wessler et al. 2011). Translocated CagA during *H. pylori* infection alters SHP-2 (SH2-containing tyrosine phosphatase 2), Crk (C-terminal Src kinase), Grb2 (growth factor receptor-bound protein 2), and MARK2 (microtubule affinity-regulating kinase 2), which dysregulate key cellular biochemical pathways, apoptosis, and rearrangements of the host actin-cytoskeleton (Tohidpour et al. 2017). Similarly, it has been described actin-rearrangement in the pancreas, during both, pancreatitis and pancreatic cancer (Morris and Machesky 2015). In this work, in the stomach of infected gerbils, we also observed important rearrangements in the cytoskeleton, as well as a reduction in the amount of filamentous actin, which should be confirmed by western blot experiments in the future. Similar results were obtained in the pancreatic tissue, where reorganization of the actin-cytoskeleton was observed in infected gerbils (Figs. 5 and 6). These changes might represent the highly variable actin dynamics, trying to maintain the homeostasis of both, acini and beta cells.

In order to regulate the release of hormones in the Langerhans islet, the endocrine cells interact with one another either through homotypic or heterotypic cell–cell adhesion or in a paracrine manner (Jain and Lammert 2009). Pancreatitis can lead to diabetes *mellitus*, where loss of functional or structural β cells and the altered insulin secretion produced by harmed junctional proteins have been described (Singh et al. 2022). Furthermore, pancreatic inflammation leads to the decrease of islet cell mass leading to the loss of glucagon, insulin, and pancreatic polypeptide, which difficult the control of diabetes with large variations in blood glucose (Singh et al. 2022). In this work, we observed that the *H. pylori* infection produced alterations in the localization pattern of insulin and glucagon, and an apparent reduction in their levels at pancreas (Fig. 7). This effect could be a consequence of the modified intercellular junction proteins. For example, claudin 4 is involved in regulating the functional state of islet, and may act as a biomarker of β‐cell maturation (Li et al. 2020). Furthermore, E-cadherin plays an important role in islet formation, glucose-stimulated insulin secretion and gap junction communication (Jain and Lammert 2009). However, more experiments should be carried out in order to demonstrate the participation of junctional proteins in the endocrine altered functions of the pancreas during *H. pylori* infection.

During host infection, IL-8 increases in response to *H. pylori*, and it is a key chemokine in accumulating neutrophils. Expression of this cytokine is often regulated by NF-κB (transcription factor complex nuclear factor-κB), through κB-binding elements in the enhancer/promoter regions of their genes (Eftang et al. 2012). Here, we confirmed that infected animals presented increased levels of IL-8 in serum (Fig. 8). This finding supports the idea that IL-8 appears paramount in the inflammatory response to *H. pylori* infection, affecting the whole organism and other organs besides the stomach, where the induced inflammation importantly contributes to the damage in pancreas.

## Conclusion

We conclude that *H. pylori* is capable to reach the pancreas, where it produces an important damage in paracellular proteins and actin-cytoskeleton, affecting the insulin and glucagon distribution. Nevertheless, the mechanisms underlying the arrival of this bacterium or its virulence factors (such as CagA or OMPs) to this secretory epithelium, should be extensively studied in future works, to better understand its relationship with pancreatic diseases.

### Supplementary Information

Below is the link to the electronic supplementary material.

Supplementary file1 (TIF 4668 KB)

Supplementary file2 (TIF 6269 KB)

Supplementary file3 (TIF 5825 KB)

Supplementary file4 (TIF 14583 KB)

Supplementary file5 (TIF 7877 KB)

Supplementary file6 (TIF 425 KB)

Supplementary file7 (DOCX 18 KB)

## Data Availability

The datasets used and/or analysed during the current study available from the corresponding author on reasonable request.

## References

[CR1] Ahmed N, Ali Khan MS, Mat Jais AM et al (2013) Anti-ulcer activity of sandalwood (Santalum album L.) stem hydro-alcoholic extract in three gastric-ulceration models of wistar rats. Bol Latinoam y Del Caribe Plantas Med y Aromat 12:81–91

[CR2] Alzahrani S, Lina TT, Gonzalez J et al (2014) Effect of *Helicobacter pylori* on gastric epithelial cells. World J Gastroenterol 20:12767–12780. 10.3748/wjg.v20.i36.12767 25278677 10.3748/wjg.v20.i36.12767 PMC4177462

[CR3] Ansari S, Yamaoka Y (2022) Animal models and *Helicobacter pylori* infection. J Clin Med 11:1–19. 10.3390/jcm11113141 10.3390/jcm11113141 PMC9181647 35683528

[CR4] Bernegger S, Vidmar R, Fonovic M et al (2021) Identification of desmoglein-2 as a novel target of *Helicobacter pylori* HtrA in epithelial cells. Cell Commun Signal 19:1–12. 10.1186/s12964-021-00788-x 34742300 10.1186/s12964-021-00788-x PMC8571890

[CR5] Bulajic M, Panic N, Löhr JM (2014) *Helicobacter pylori* and pancreatic disease. World J Gastrointest Pathophysiol 5:380–383. 10.4291/wjgp.v5.i4.380 25400980 10.4291/wjgp.v5.i4.380 PMC4231501

[CR6] Chauhan N, Tay ACY, Marshall BJ, Jain U (2019) *Helicobacter pylori* VacA, a distinct toxin exerts diverse functionalities in numerous cells: an overview. Helicobacter 24:1–9. 10.1111/hel.12544 10.1111/hel.12544 30324717

[CR7] Chen Y, Wang X, Yu Y et al (2018) Serum exosomes of chronic gastritis patients infected with *Helicobacter pylori* mediate IL-1α expression via IL-6 trans-signalling in gastric epithelial cells. Clin Exp Immunol 194:339–349. 10.1111/cei.13200 30105789 10.1111/cei.13200 PMC6231010

[CR8] Chun MGH, Hanahan D (2010) Genetic deletion of the desmosomal component Desmoplakin promotes tumor microinvasion in a mouse model of pancreatic neuroendocrine carcinogenesis. PLoS Genet. 10.1371/journal.pgen.1001120 20862307 10.1371/journal.pgen.1001120 PMC2940733

[CR9] Cortés-Márquez AC, Mendoza-Elizalde S, Arenas-Huertero F et al (2018) Differential expression of miRNA-146a and miRNA-155 in gastritis induced by *Helicobacter pylori* infection in paediatric patients, adults, and an animal model. BMC Infect Dis 18:1–9. 10.1186/s12879-018-3368-2 30219037 10.1186/s12879-018-3368-2 PMC6139157

[CR10] Costa AM, Leite M, Seruca R, Figueiredo C (2013) Adherens junctions as targets of microorganisms: a focus on *Helicobacter pylori*. FEBS Lett 587:259–265. 10.1016/j.febslet.2012.12.008 23262219 10.1016/j.febslet.2012.12.008

[CR11] De Kort S, Keszthelyi D, Masclee AAM (2011) Leaky gut and diabetes mellitus: what is the link? Obes Rev 12:449–458. 10.1111/j.1467-789X.2010.00845.x 21382153 10.1111/j.1467-789X.2010.00845.x

[CR12] Di J, Zhang J, Cao L et al (2020) Hydrogen peroxide-mediated oxygen enrichment eradicates *Helicobacter pylori* in vitro and in vivo. Antimicrob Agents Chemother 64:1–15. 10.1128/AAC.02192-19 10.1128/AAC.02192-19 PMC7179623 32071054

[CR13] Doohan D, Rezkitha YAA, Waskito LA et al (2021) *Helicobacter pylori* baba–saba key roles in the adherence phase: The synergic mechanism for successful colonization and disease development. Toxins (Basel). 10.3390/toxins13070485 34357957 10.3390/toxins13070485 PMC8310295

[CR14] Dore MP, Sepulveda AR, Pedroni A et al (2008) Reversal of elevated pancreatic enzymes after *Helicobacter pylori* eradication. Intern Emerg Med 3:269–270. 10.1007/s11739-008-0117-3 18264669 10.1007/s11739-008-0117-3

[CR15] Eftang LL, Esbensen Y, Tannæs TM et al (2012) Interleukin-8 is the single most up-regulated gene in whole genome profiling of H. pylori exposed gastric epithelial cells. BMC Microbiol 12:1–15. 10.1186/1471-2180-12-9 22248188 10.1186/1471-2180-12-9 PMC3292955

[CR16] Espinoza MGC, Vazquez RG, Mendez IM et al (2011) Detection of the glmm gene in *Helicobacter pylori* isolates with a novel primer by PCR. J Clin Microbiol 49:1650–1652. 10.1128/JCM.00461-10 21289140 10.1128/JCM.00461-10 PMC3122814

[CR17] Gravina AG, Priadko K, Ciamarra P et al (2020) Extra-gastric manifestations of *Helicobacter pylori* infection. J Clin Med 9:3887. 10.3390/jcm9123887 33265933 10.3390/jcm9123887 PMC7761397

[CR18] Heimesaat MM, Fischer A, Plickert R et al (2014) *Helicobacter pylori* induced gastric immunopathology is associated with distinct microbiota changes in the large intestines of long-term infected Mongolian gerbils. PLoS ONE 9:1–11. 10.1371/journal.pone.0100362 10.1371/journal.pone.0100362 PMC4062524 24941045

[CR19] Hooi JKY, Lai WY, Ng WK et al (2017) Global prevalence of *Helicobacter pylori* infection: systematic review and meta-analysis. Gastroenterology 153:420–429. 10.1053/j.gastro.2017.04.022 28456631 10.1053/j.gastro.2017.04.022

[CR20] Jain R, Lammert E (2009) Cell-cell interactions in the endocrine pancreas. Diabetes Obes Metab 11:159–167. 10.1111/j.1463-1326.2009.01102.x 19817798 10.1111/j.1463-1326.2009.01102.x

[CR21] Jarzab M, Posselt G, Meisner-Kober N, Wessler S (2020) *Helicobacter pylori*-derived outer membrane vesicles (OMVs): role in bacterial pathogenesis? Microorganisms 8:1328. 10.3390/microorganisms8091328 32878302 10.3390/microorganisms8091328 PMC7564109

[CR22] Jeyamani L, Jayarajan J, Leelakrishnan V, Swaminathan M (2018) CagA and VacA genes of *Helicobacter pylori* and their clinical relevance. Indian J Pathol Microbiol 61:66. 10.4103/IJPM.IJPM_234_17 29567886 10.4103/IJPM.IJPM_234_17

[CR23] Jimenez-Caliani AJ, Pillich R, Yang W et al (2017) αE-Catenin is a positive regulator of pancreatic islet cell lineage differentiation. Cell Rep 20:1295–1306. 10.1016/j.celrep.2017.07.035 28793255 10.1016/j.celrep.2017.07.035 PMC5611824

[CR24] Kojima T, Yamaguchi H, Ito T et al (2013) Tight junctions in human pancreatic duct epithelial cells. Tissue Barriers 1:e24894. 10.4161/tisb.24894 24665406 10.4161/tisb.24894 PMC3805649

[CR25] Kunovsky L, Dite P, Jabandziev P et al (2021) *Helicobacter pylori* infection and other bacteria in pancreatic cancer and autoimmune pancreatitis. World J Gastrointest Oncol 13:835–844. 10.4251/wjgo.v13.i8.835 34457189 10.4251/wjgo.v13.i8.835 PMC8371525

[CR26] Lage AP, Godfroid E, Fauconnier A et al (1995) Diagnosis of *Helicobacter pylori* infection by PCR: comparison with other invasive techniques and detection of cagA gene in gastric biopsy specimens. J Clin Microbiol 33:2752–2756. 10.1128/jcm.33.10.2752-2756.1995 8567918 10.1128/jcm.33.10.2752-2756.1995 PMC228568

[CR27] Lertsethtakarn P, Ottemann KM, Hendrixson DR (2011) Motility and chemotaxis in campylobacter and helicobacter. Annu Rev Microbiol 65:389–410. 10.1146/annurev-micro-090110-102908 21939377 10.1146/annurev-micro-090110-102908 PMC6238628

[CR28] Li H, Neelankal John A, Nagatake T et al (2020) Claudin 4 in pancreatic β cells is involved in regulating the functional state of adult islets. FEBS Open Bio 10:28–40. 10.1002/2211-5463.12735 31562747 10.1002/2211-5463.12735 PMC6943228

[CR29] Li X, Lu J, Wang Y et al (2016) Establishment and characterization of a newly established diabetic Gerbil line. PLoS ONE 11:1–13. 10.1371/journal.pone.0159420 10.1371/journal.pone.0159420 PMC4948894 27427908

[CR30] Honda S, Fujioka T, Tokieda M, Gotoh T, Nishizono A, Nasu M (1998) Gastric ulcer, atrophic gastritis, and intestinal metaplasia caused by *Helicobacter pylori* infection in mongolian gerbils. Scand J Gastroenterol 33:454–460. 10.1080/00365529850171990 9648982 10.1080/00365529850171990

[CR31] Malfertheiner P, Camargo MC, El-Omar E et al (2023) *Helicobacter pylori* infection. Nat Rev Dis Prim. 10.1038/s41572-023-00431-8 37081005 10.1038/s41572-023-00431-8 PMC11558793

[CR32] Manes G, Balzano A, Vaira D (2003) *Helicobacter pylori* and pancreatic disease. JOP 4:111–116 12743416

[CR33] Matsuo Y, Kido Y, Yamaoka Y (2017) *Helicobacter pylori* outer membrane protein-related pathogenesis. Toxins (Basel) 9:1–9. 10.3390/toxins9030101 10.3390/toxins9030101 PMC5371856 28287480

[CR34] Mendoza-Elizalde S, Arteaga-resendiz NK, Valencia-mayoral P, Velázquez-guadarrama N (2016) Diversification of the vacAs1m1 and vacAs2m2 Strains of *Helicobacter pylori* in Meriones unguiculatus. Front Microbiol 7:1–11. 10.3389/fmicb.2016.01758 27877163 10.3389/fmicb.2016.01758 PMC5100360

[CR35] Mobley HLT (2001) Urease. In: Achtman MSS (ed) *Helicobacter pylori*: physiology and genetics. Horizon Scientific Press, Wymondham, pp 155–710

[CR36] Morris HT, Machesky LM (2015) Actin cytoskeletal control during epithelial to mesenchymal transition: focus on the pancreas and intestinal tract. Br J Cancer 112:613–620. 10.1038/bjc.2014.658 25611303 10.1038/bjc.2014.658 PMC4333498

[CR37] Myo Min KK, Rojas-Canales D, Penko D et al (2022) Desmoglein-2 is important for islet function and β-cell survival. Cell Death Dis 13:1–15. 10.1038/s41419-022-05326-2 10.1038/s41419-022-05326-2 PMC9617887 36309486

[CR38] Nilsson H-O, Stenram U, Ihse I, Wadstrom T (2006) Helicobacter species ribosomal DNA in the pancreas, stomach and duodenum of pancreatic cancer patients. World J Gastroenterol 12:3038–3043. 10.3748/wjg.v12.i19.3038 16718784 10.3748/wjg.v12.i19.3038 PMC4124378

[CR39] Ohkusa T, Okayasu I, Miwa H et al (2003) *Helicobacter pylori* infection induces duodenitis and superficial duodenal ulcer in Mongolian gerbils. Gut 52:797–803. 10.1136/gut.52.6.797 12740333 10.1136/gut.52.6.797 PMC1773688

[CR40] Pandol SJ (2017) The exocrine pancreas. Morgan & Claypool Life Sciences All 21634067

[CR41] Pellicano R, Ménard A, Rizzetto M, Mégraud F (2008) Helicobacter species and liver diseases: association or causation? Lancet Infect Dis 8:254–260. 10.1016/S1473-3099(08)70066-5 18353266 10.1016/S1473-3099(08)70066-5

[CR42] Polyzos SA, Papaefthymiou A, Doulberis M et al (2021) *Helicobacter pylori* infection and diabetes mellitus. Diabetes Metab Syndr Clin Res Rev 15:845–846. 10.1016/j.dsx.2021.03.034 10.1016/j.dsx.2021.03.034 33873053

[CR43] Rabelo-Gonçalves EM, Roesler BM, Zeitune JM (2015) Extragastric manifestations of *Helicobacter pylori* infection: possible role of bacterium in liver and pancreas diseases. World J Hepatol 7:2968–2979. 10.4254/wjh.v7.i30.2968 26730276 10.4254/wjh.v7.i30.2968 PMC4691700

[CR44] Rieder G, Karnholz A, Stoeckelhuber M et al (2007) H pylori infection causes chronic pancreatitis in Mongolian gerbils. World J Gastroenterol 13:3939–3947. 10.3748/wjg.v13.i29.3939 17663507 10.3748/wjg.v13.i29.3939 PMC4171165

[CR45] Rocha M, Avenaud P, Ménard A et al (2005) Association of Helicobacter species with hepatitis C cirrhosis with or without hepatocellular carcinoma. Gut 54:396–401. 10.1136/gut.2004.042168 15710989 10.1136/gut.2004.042168 PMC1774397

[CR46] Röder PV, Wu B, Liu Y, Han W (2016) Pancreatic regulation of glucose homeostasis. Exp Mol Med 48:e219. 10.1038/emm.2016.6 26964835 10.1038/emm.2016.6 PMC4892884

[CR47] Sato T, Shibata W, Maeda S (2019) Adhesion molecules and pancreatitis. J Gastroenterol 54:99–107. 10.1007/s00535-018-1500-0 30140950 10.1007/s00535-018-1500-0 PMC6349808

[CR48] Serrill JD, Sander M, Shih HP (2018) Pancreatic exocrine tissue architecture and integrity are maintained by E-cadherin during postnatal development. Sci Rep 8:1–12. 10.1038/s41598-018-31603-2 30194315 10.1038/s41598-018-31603-2 PMC6128895

[CR49] Sharma SA, Tummuru MKR, Blaser MJ, Kerr LD (1998) Activation of IL-8 gene expression by *Helicobacter pylori* is regulated by transcription factor nuclear factor-κB in gastric epithelial cells. J Immunol 160:2401–2407. 10.4049/jimmunol.160.5.2401 9498783 10.4049/jimmunol.160.5.2401

[CR50] Shojaee Tabrizi A, Derakhshandeh A, Esfandiari A, Ali Atashi Z (2015) Identification of Helicobacter spp. in gastrointestinal tract, pancreas and hepatobiliary system of stray cats. Iran J Vet Res 16:374–376 27175206 PMC4782678

[CR51] Singh A, Aggarwal M, Garg R et al (2022) Post-pancreatitis diabetes mellitus: insight on optimal management with nutrition and lifestyle approaches. Ann Med 54:1776–1786. 10.1080/07853890.2022.2090601 35786076 10.1080/07853890.2022.2090601 PMC9254994

[CR52] Smith S (2004) Comparison of three PCR methods for detection of *Helicobacter pylori* DNA and detection of cagA gene in gastric biopsy specimens. World J Gastroenterol 10:1958. 10.3748/wjg.v10.i13.1958 15222045 10.3748/wjg.v10.i13.1958 PMC4572239

[CR53] Song X, Cai C, Jin Q et al (2021) The efficacy of *Helicobacter pylori* eradication in diabetics and its effect on glycemic control: a systematic review and meta-analysis. Helicobacter 26:1–15. 10.1111/hel.12781 10.1111/hel.12781 33465265

[CR54] Suerbaum S, Michetti P (2002) *Helicobacter pylori* infection. N Engl J Med 347:1175–1186. 10.1056/NEJMra020542 12374879 10.1056/NEJMra020542

[CR55] Takahashi-Kanemitsu A, Knight CT, Hatakeyama M (2020) Molecular anatomy and pathogenic actions of *Helicobacter pylori* CagA that underpin gastric carcinogenesis. Cell Mol Immunol 17:50–63. 10.1038/s41423-019-0339-5 31804619 10.1038/s41423-019-0339-5 PMC6952403

[CR56] Tegtmeyer N, Wessler S, Necchi V et al (2017) *Helicobacter pylori* employs a unique basolateral type IV secretion mechanism for CagA delivery. Cell Host Microbe 22:552-560.e5. 10.1016/j.chom.2017.09.005 29024645 10.1016/j.chom.2017.09.005

[CR57] Testerman TL, Morris J (2014) Beyond the stomach: an updated view of *Helicobacter pylori* pathogenesis, diagnosis, and treatment. World J Gastroenterol 20:12781–12808. 10.3748/wjg.v20.i36.12781 25278678 10.3748/wjg.v20.i36.12781 PMC4177463

[CR58] Tohidpour A, Gorrell RJ, Roujeinikova A, Kwok T (2017) The middle fragment of *Helicobacter pylori* CagA induces Actin rearrangement and triggers its own uptake into gastric epithelial cells. Toxins (Basel) 9:1–16. 10.3390/toxins9080237 10.3390/toxins9080237 PMC5577571 28788072

[CR59] Velazquez-Guadarrama N, Olivares A, Valencia P et al (2007) Genotoxic and oxidative damage induced by *Helicobacter pylori* in Meriones unguiculatus. J Environ Pathol Toxicol Oncol 26:39–49. 10.1615/JEnvironPatholToxicolOncol.v26.i1.50 17725529 10.1615/JEnvironPatholToxicolOncol.v26.i1.50

[CR60] Wessler S, Gimona M, Rieder G (2011) Regulation of the actin cytoskeleton in *Helicobacter pylori*-induced migration and invasive growth of gastric epithelial cells. Cell Commun Signal 9:1–9. 10.1186/1478-811X-9-27 22044652 10.1186/1478-811X-9-27 PMC3214149

[CR61] Xu C, Soyfoo DM, Wu Y, Xu S (2020) Virulence of *Helicobacter pylori* outer membrane proteins: an updated review. Eur J Clin Microbiol Infect Dis 39:1821–1830. 10.1007/s10096-020-03948-y 32557327 10.1007/s10096-020-03948-y PMC7299134

[CR62] Zhao YR, Zhou Y, Lin G et al (2015) Association between IL-17, IL-8 and IL-18 expression in peripheral blood and *Helicobacter pylori* infection in mongolian gerbils. Jundishapur J Microbiol. 10.5812/jjm.21503 26464765 10.5812/jjm.21503 PMC4600202

