# Supplementary figures and images for: The Helicobacter pylori infection alters the intercellular junctions on the pancreas of gerbils (Meriones unguiculatus)
Source: World J Microbiol Biotechnol. 2024 Jul 20;40(9):273. doi: 10.1007/s11274-024-04081-0 (PMC11271430; doi:10.1007/s11274-024-04081-0)

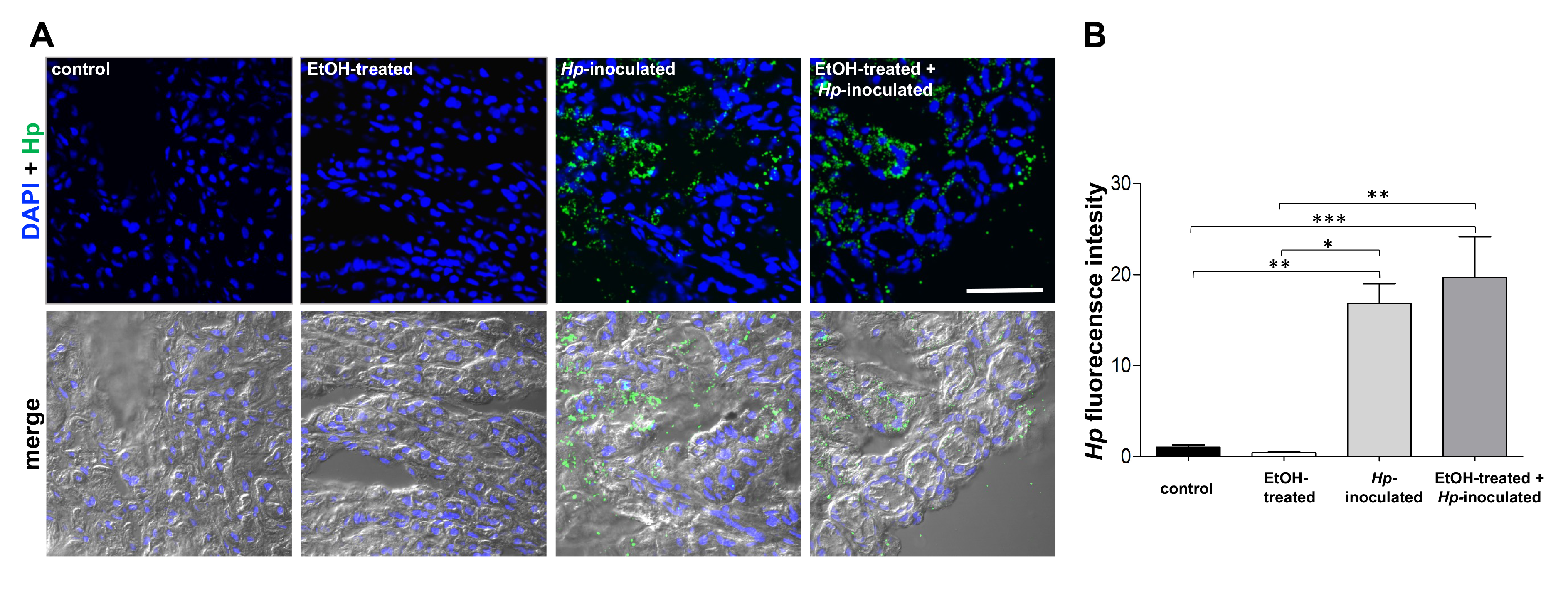

Supplement: Supplementary file 1 — Supplementary file1 (TIF 4668 KB) [file 11274_2024_4081_MOESM1_ESM.tif]

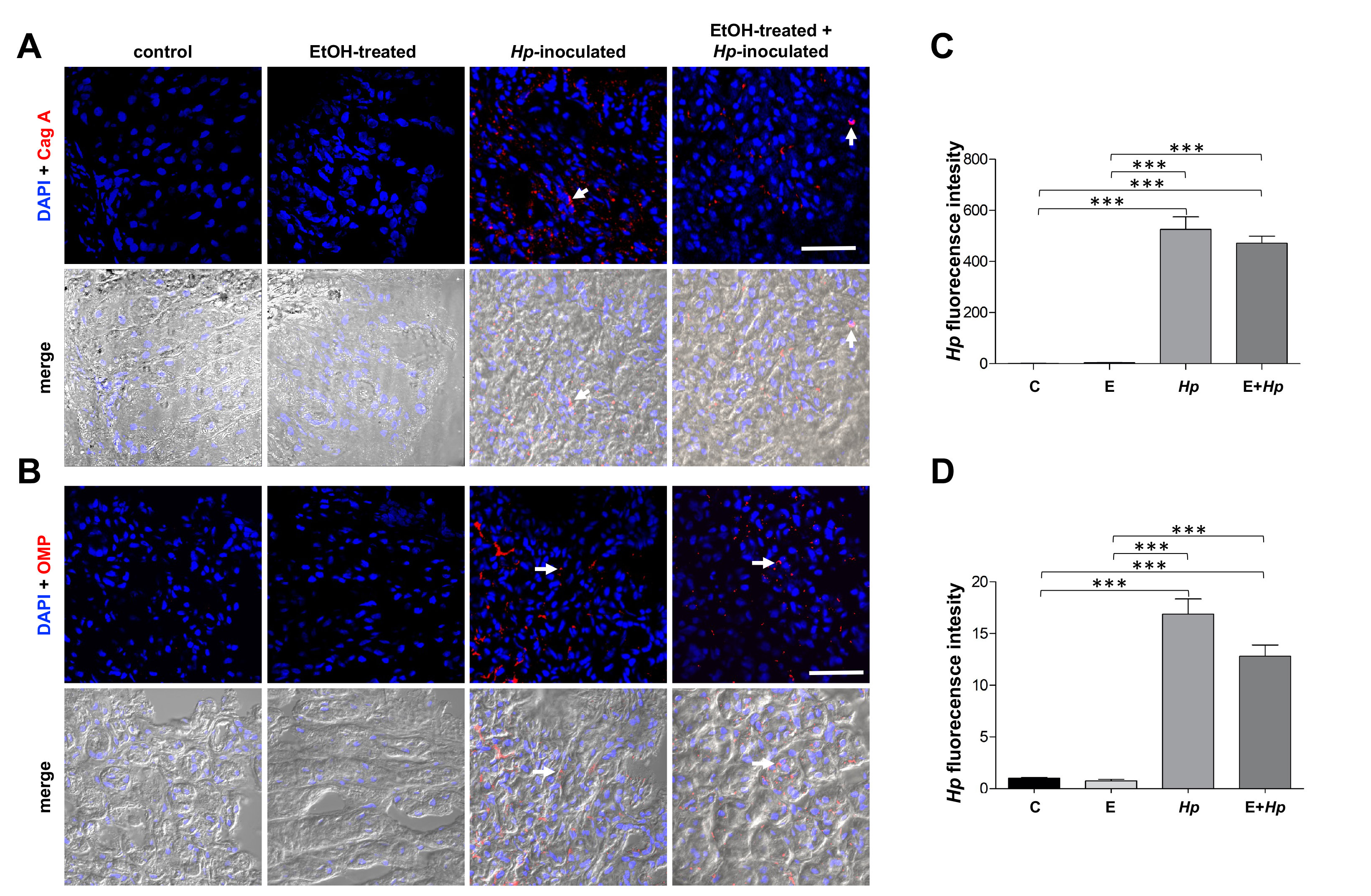

Supplement: Supplementary file 2 — Supplementary file2 (TIF 6269 KB) [file 11274_2024_4081_MOESM2_ESM.tif]

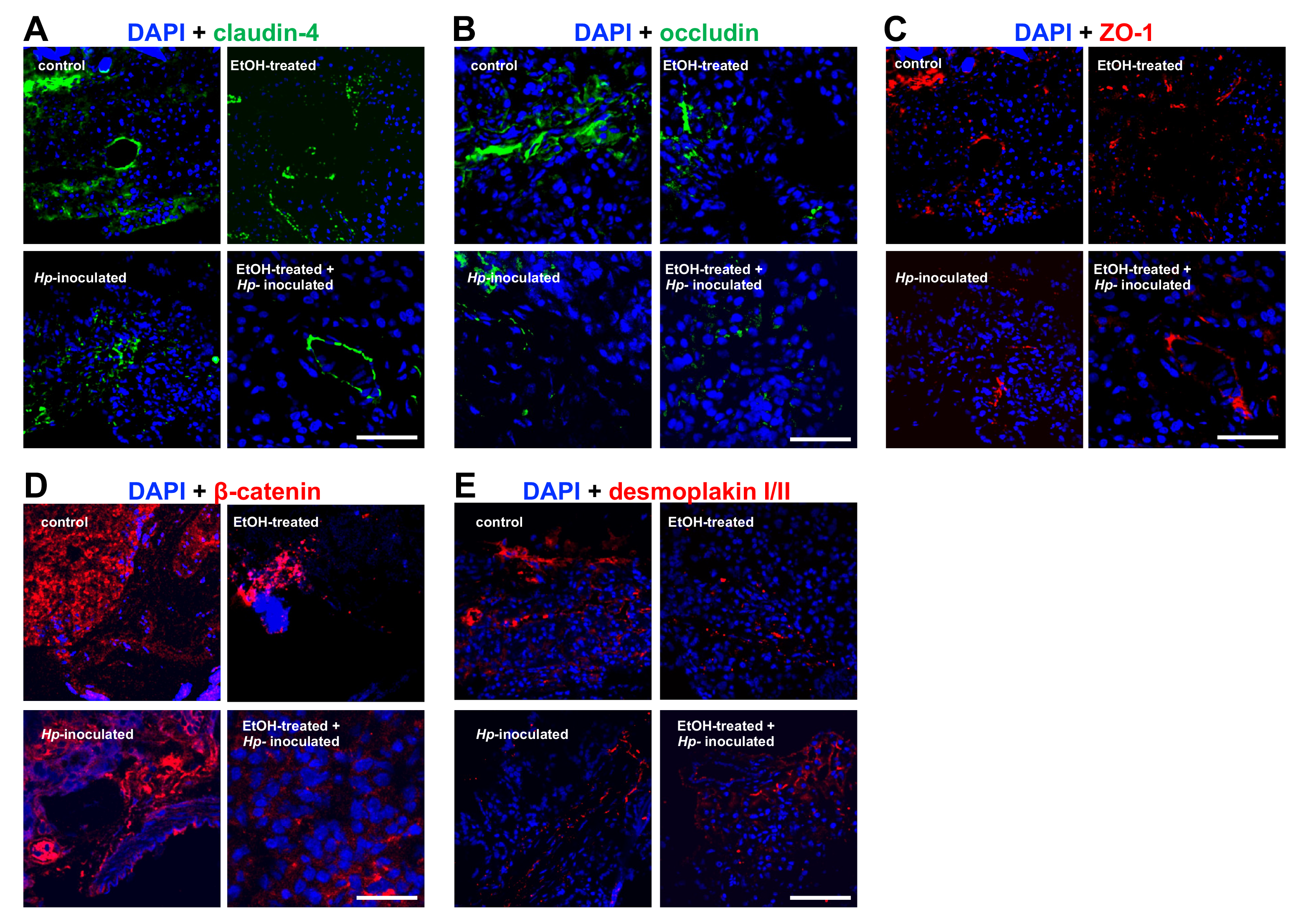

Supplement: Supplementary file 3 — Supplementary file3 (TIF 5825 KB) [file 11274_2024_4081_MOESM3_ESM.tif]

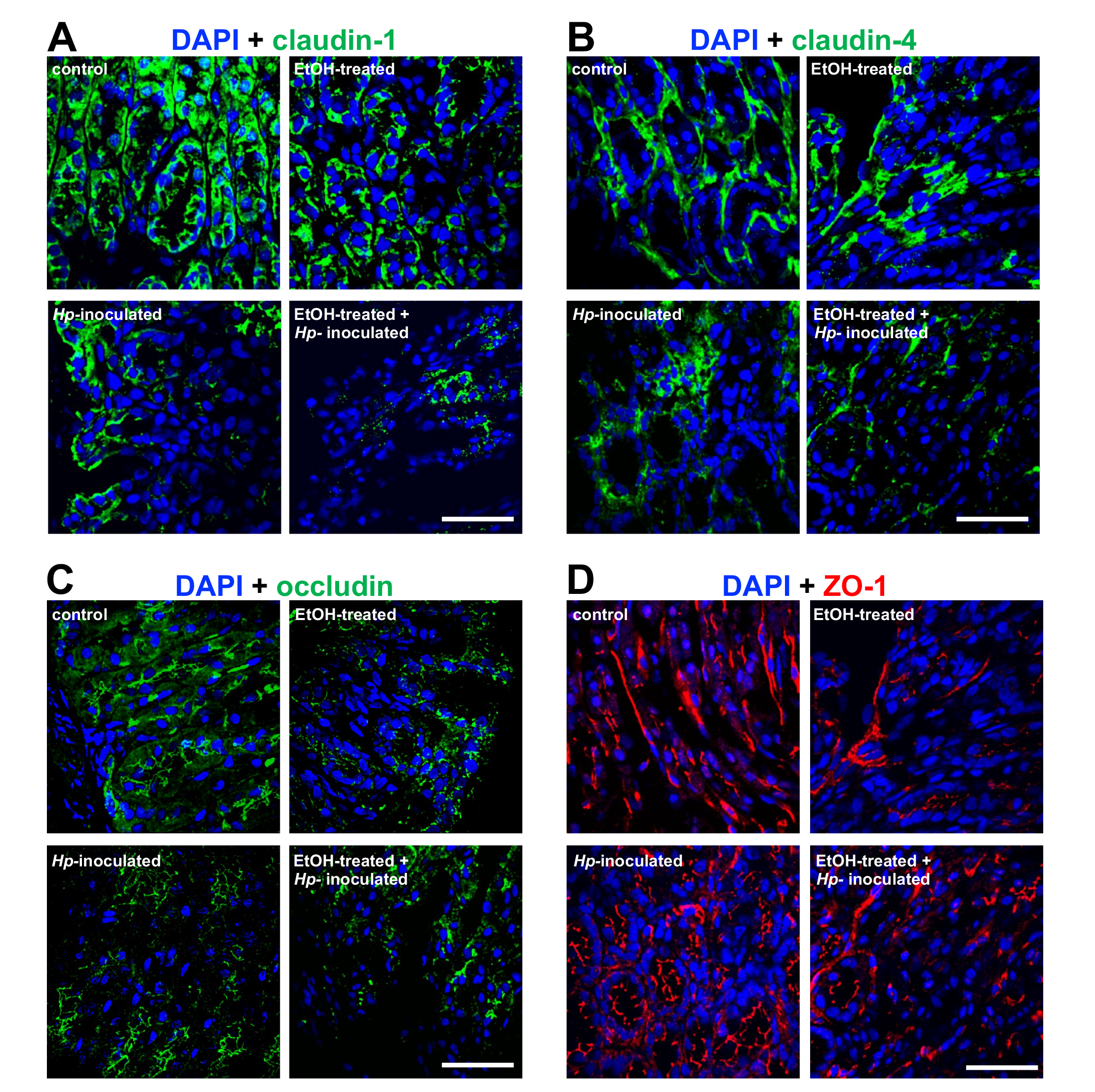

Supplement: Supplementary file 4 — Supplementary file4 (TIF 14583 KB) [file 11274_2024_4081_MOESM4_ESM.tif]

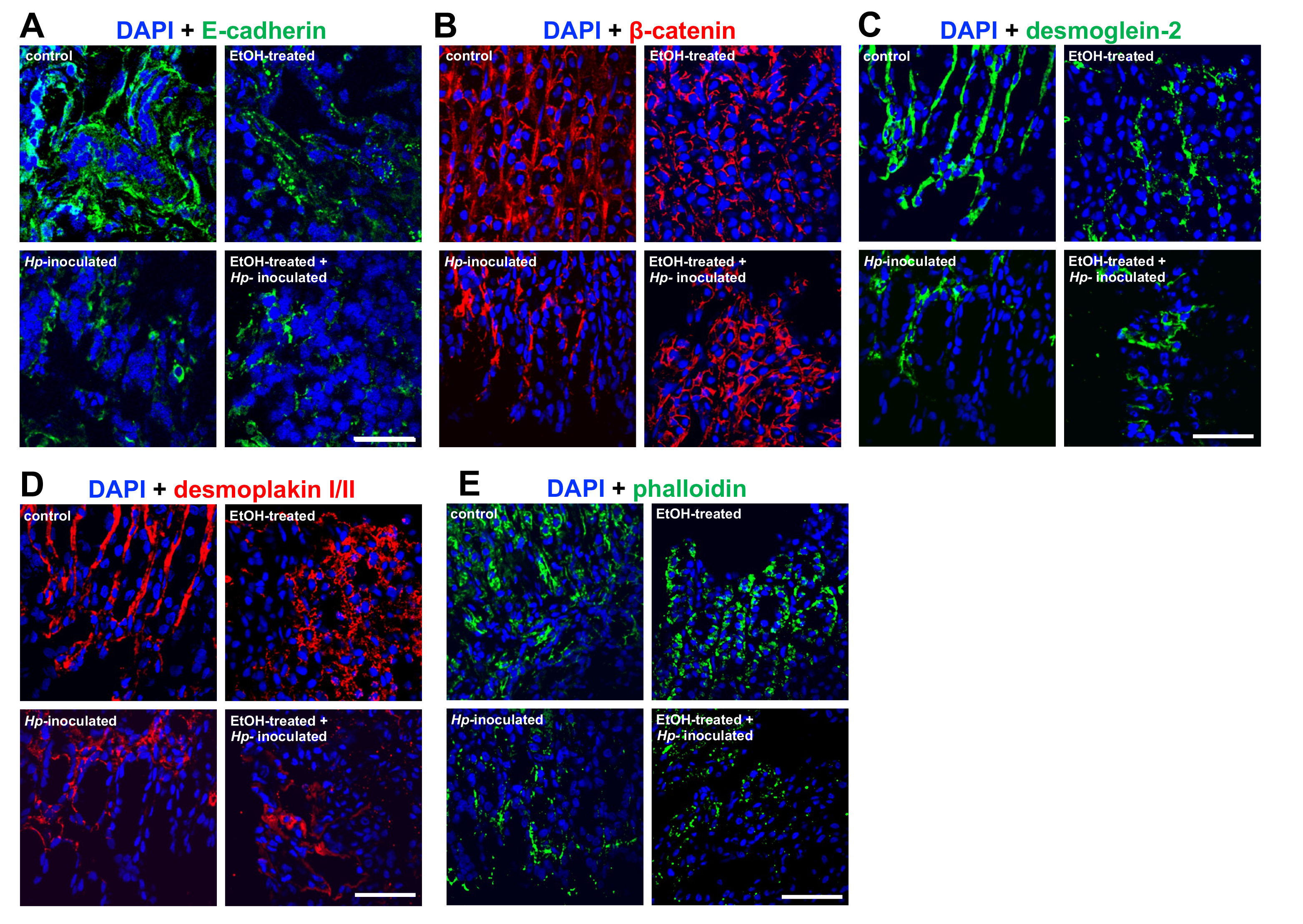

Supplement: Supplementary file 5 — Supplementary file5 (TIF 7877 KB) [file 11274_2024_4081_MOESM5_ESM.tif]

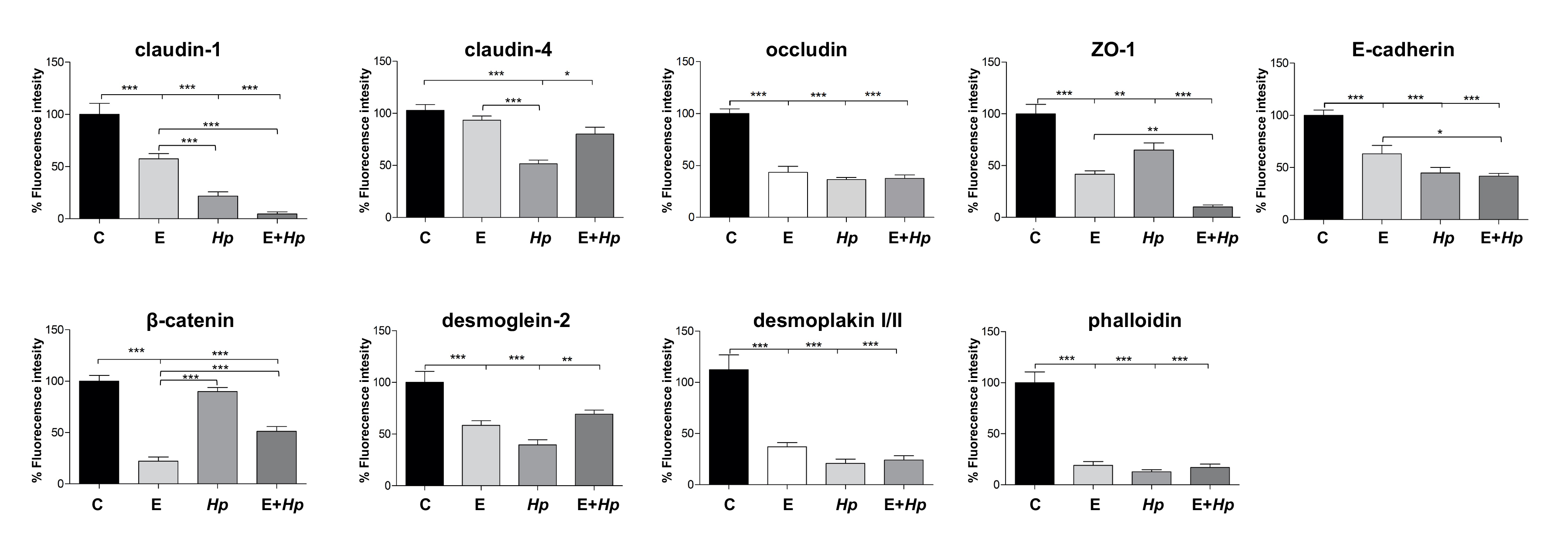

Supplement: Supplementary file 6 — Supplementary file6 (TIF 425 KB) [file 11274_2024_4081_MOESM6_ESM.tif]
