# Supplementary material for: The Helicobacter pylori infection alters the intercellular junctions on the pancreas of gerbils (Meriones unguiculatus)
Source: World J Microbiol Biotechnol. 2024 Jul 20;40(9):273. doi: 10.1007/s11274-024-04081-0 (PMC11271430; doi:10.1007/s11274-024-04081-0)
Supplement: Supplementary file 7 — Supplementary file7 (DOCX 18 KB) [file 11274_2024_4081_MOESM7_ESM.docx]

***World Journal of Microbiology and Biotechnology***

**The *Helicobacter pylori* infection alters the intercellular junctions on the pancreas of gerbils (*Meriones unguiculatus*)**

Edgar G. Hurtado-Monzón^1^ (ORCID 0009-0004-4055-3678), Pedro Valencia-Mayoral^2^ (ORCID 0009-0008-5979-6652), Angélica Silva-Olivares^1^, Cecilia Bañuelos^3^ (ORCID 0000-0002-8168-4488), Norma Velázquez-Guadarrama^4,*^ (ORCID 0000-0002-3620-7260), Abigail Betanzos^1,*^ (ORCID 0000-0003-1761-0481)

^1^ Departamento de Infectómica y Patogénesis Molecular, Centro de Investigación y de Estudios Avanzados del Instituto Politécnico Nacional (CINVESTAV-IPN), Ciudad de México, México

^2^ Departamento de Patología Clínica y Experimental del Hospital Infantil de México Federico Gómez, Ciudad de México, México

^3^ Programa de Doctorado Transdisciplinario en Desarrollo Científico y Tecnológico para la Sociedad, CINVESTAV-IPN, Ciudad de México, México

^4^ Laboratorio de Investigación en Enfermedades Infecciosas, Área de Genética Bacteriana del Hospital Infantil de México Federico Gómez, Ciudad de México, México

*Corresponding authors: abetanzos@cinvestav.mx (AB) and normave@himfg.edu.mx (NVG)

**Supporting information**

**S1 Fig. Presence of *H. pylori* at gerbil stomach.** **A)** Stomach sections were processed for immunofluorescence assays using ⍺-*Hp* antibody (green). Nuclei (blue) were stained with DAPI. Merge: fluorescence images overlapped with the phase-contrast bright field. Bar = 50 μm. Representative images from one animal are shown for each group (n = 3). **B)** Green fluorescence intensity was measured by pixels using the ImageJ software. All groups were statistically compared regarding control and EtOH-treated animals. (*) p<0.05, (**) p<0.01 and (***) p<0.001.

**S2 Fig. Presence of *H. pylori* proteins at gerbil stomach**. **A,B)** Stomach sections were processed for immunofluorescence assays using ⍺-CagA (**A**) or ⍺-OMP (**B**) antibodies (red). Nuclei (blue) were stained with DAPI. Merge: fluorescence images overlapped with the phase-contrast bright field. Bar = 50 μm. Representative images from one animal are shown for each group (n = 3). **C,D)** Red fluorescence intensity of CagA (**C**) or OMP (**D**) was measured by pixels using the ImageJ software. All groups were statistically compared regarding control and EtOH-treated animals. (***) *p*<0.001. C: control; E: EtOH-treated; *Hp*: *Hp-*inoculated; E+*Hp*: EtOH-treated plus *Hp-*inoculated.

**S3 Fig. Alterations of intercellular junction proteins at gerbil pancreas by *H. pylori* infection. A-E)** Pancreas sections were processed for immunofluorescence assays using ⍺-claudin-4 **(A),** ⍺-occludin **(B)** (green)**,** ⍺-ZO-1 **(C),** ⍺-β-catenin **(D)** or ⍺-desmoplakin I/II **(E)** antibodies (red). Nuclei (blue) were DAPI stained. Bar = 50 μm. Representative images from one animal are shown for each group (n = 3).

**S4 Fig. Modifications of tight junction proteins at gerbil stomach by *H. pylori* infection. A-D)** Stomach sections were processed for immunofluorescence assays using ⍺-claudin-1 **(A)** (green)**,** ⍺-claudin-4 **(B)** (green), ⍺-occludin **(C)** (green) or ⍺-ZO-1 **(D)** (red) antibodies. Nuclei (blue) were stained with DAPI. Bar = 50 μm. Representative images from one animal are shown for each group (n = 3).

**S5 Fig. Alterations of intercellular junction proteins at gerbil stomach by *H. pylori* infection. A-D)** Pancreas sections were processed for immunofluorescence assays using ⍺-E-cadherin (green) **(A),** ⍺-β-catenin (red) **(B),** ⍺-desmoglein-2 (green) **(C)** or α-desmoplakin I/II (red) antibodies. **E)** polymerized actin was stained with phalloidin-FITC (green). Nuclei (blue) were stained with DAPI. Bar = 50 μm. Representative images from one animal are shown for each group (n = 3).

**S6 Fig. Levels of intercellular junction proteins at gerbil stomach by *H. pylori* infection.** Fluorescence intensity of occludin, claudin-1, claudin-4, ZO-1, E-cadherin, β-catenin, desmoglein-2, desmoplakin I/II and phalloidin was measured by pixels using the ImageJ software. All groups were statistically compared regarding control and EtOH-treated animals. (*) *p*<0.05, (**) *p*<0.01 and (***) *p*<0.001. C: control; E: EtOH-treated; *Hp*: *Hp-*inoculated; E+*Hp*: EtOH-treated plus *Hp-*inoculated. n=3
